# PGC-1α Is a Master Regulator of Mitochondrial Lifecycle and ROS Stress Response

**DOI:** 10.3390/antiox12051075

**Published:** 2023-05-10

**Authors:** Othman Abu Shelbayeh, Tasnim Arroum, Silke Morris, Karin B. Busch

**Affiliations:** 1 Institute of Integrative Cell Biology and Physiology, University of Münster, Schlossplatz 5, 48149 Münster, Germany; 2 Molecular Medicine and Genetics, Wayne State University, Detroit, MI 48202, USA

**Keywords:** PGC-1α, ROS defense, mitonuclear communication, mitochondrial life cycle

## Abstract

Mitochondria play a major role in ROS production and defense during their life cycle. The transcriptional activator PGC-1α is a key player in the homeostasis of energy metabolism and is therefore closely linked to mitochondrial function. PGC-1α responds to environmental and intracellular conditions and is regulated by SIRT1/3, TFAM, and AMPK, which are also important regulators of mitochondrial biogenesis and function. In this review, we highlight the functions and regulatory mechanisms of PGC-1α within this framework, with a focus on its involvement in the mitochondrial lifecycle and ROS metabolism. As an example, we show the role of PGC-1α in ROS scavenging under inflammatory conditions. Interestingly, PGC-1α and the stress response factor NF-κB, which regulates the immune response, are reciprocally regulated. During inflammation, NF-κB reduces PGC-1α expression and activity. Low PGC-1α activity leads to the downregulation of antioxidant target genes resulting in oxidative stress. Additionally, low PGC-1α levels and concomitant oxidative stress promote NF-κB activity, which exacerbates the inflammatory response.

## 1. Introduction

The peroxisome proliferator-activated receptor γ coactivator 1α (PGC-1α is described as the master regulator of mitochondrial biogenesis and function. It was identified together with the peroxisome proliferator-activated receptor γ (PPARγ) transcription factor in mitochondria-rich and thermogenesis-specialized brown adipose tissue (BAT). Current studies suggest a role of PGC-1α in the regulation of oxidative phosphorylation (OXPHOS), fatty acid (FA)/lipid metabolism, and the modulation of reactive oxygen species (ROS). PGC-1α is found mainly in metabolically active tissues, such as the liver, kidney, skeletal muscle, brain, and adipose tissue [1,2,3]. It is also involved in the transformation of white adipose tissue into brown adipose tissue [4]. In mammals, fasting, exercise, and cold are associated with an increase in PGC-1α levels [5,6,7]. PGC-1α subsequently upregulates respiratory gene expression in the mitochondria [8].

**Figure 1:**
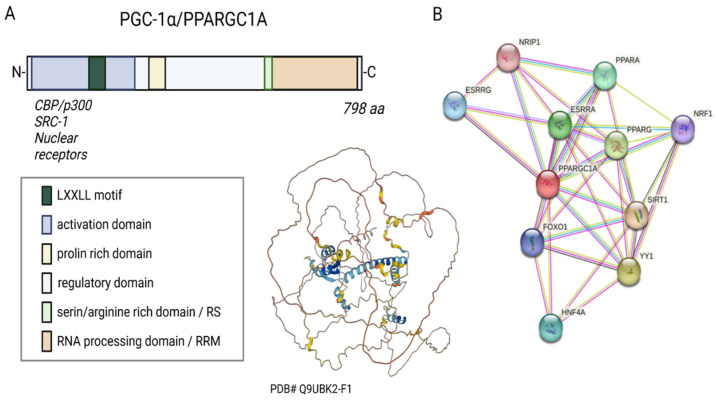
Sequence, putative structure, and interactions of PGC-1α. (**A**) Domains and structure of PGC-1α. Structure generated with α-fold [12,13]. (**B**) Known and experimental interactions of the PGC-1α/PPARGC with proteins based on the STRING database entries (https://string-db.org, accessed on 11 October 2022). Created with BioRender.com, agreement No: XW25C9KJWR.

PGC-1α belongs to the so-called PGC family of transcriptional regulators. Other members of the family are PGC-1β and PRC (PGC-1-related coactivator). The human PGC-1α consists of 798 amino acids, has a molecular weight of 91 kDa, and can be divided into several functional regions, such as the activation domain, inactivation domain, short serine/arginine-rich stretches (RS) domain, and the RNA recognition motif (RRM) [7,9] (Figure 1A). Its structure is not resolved yet.

PGC-1 family members do not have a DNA-binding domain. Also, PGC-1α does not have an intrinsic histone acetyltransferase activity, which is present in other transcriptional coactivators, that initiates gene transcription and chromatin remodeling. PGC-1α acts more as a transcriptional regulator by providing a docking platform for proteins that possess histone acetyltransferase activity. Therefore, PGC-1α indirectly promotes transcription [10,11].

Three LXXLL leucine-rich motifs (NR boxes) are located at the N-terminal of PGC-1α’s activation region and its adjacent inactive region. These can bind to several nuclear receptors, such as NR, peroxisome proliferator-activated receptor α (PPARα), estrogen receptor (ER), or nuclear respiratory factor 1 and 2 (NRF-1/NRF-2) (Figure 1B) [14,15,16]. The serine/arginine-rich domain (RS) and the RNA processing domain (RRM) motifs towards the C-terminus are typical for proteins involved in RNA splicing [17,18]. Monsalve et al. showed in in vitro studies that the C-terminal functional region participates in mRNA(messenger RNA) processing to regulate gene expression. Mutations in the RS and RRM motifs of PGC-1α affect PGC-1α’s ability to interact with transcription factors and thus impair gene transcription [19].

## 2. Regulation of PGC-1α

### 2.1. Splice Variants of PGC-1α

PGC-1α gene transcription is regulated by multiple promoter regions and is coupled with alternative splicing, resulting in a variety of PGC-1α protein variants [20]. The combination of alternative splicing and alternative use of promoters is a common process for increasing transcriptome complexity [21]. New splice variants are generated by transcription from an evolutionarily conserved alternative promoter (AP), which was found approximately 14 kb upstream of PGC-1α’s transcription start site (TSS) [22]. The transcripts of this AP contain a new exon 1 (exon 1b) with two splicing options, resulting in proteins with two different amino termini (PGC-1α-b, 12 aa long and PGC-1α-c, 3 aa long). This exon is shorter than the proximal exon 1a, which encodes for a 16 aa-long N-terminus [22,23]. The newly discovered isoforms were found in skeletal muscle after exercise [24,25,26,27] and apparently, their generation is more responsive to stimulation. Studies with PGC-1α-b in the skeletal muscle of transgenic mice revealed that a change in the mitochondrial volume is directly correlated with an improvement in exercise performance and oxidative capacity [28]. Nevertheless, the structure and function of PGC-1α-b and PGC-1α-c do not differ notably from the canonical PGC-1α [22,23].

In addition to the AP and its resulting isoforms, further TSSs and tissue-specific promoters have been described for PGC-1α in the liver, kidney, and brain. These isoforms need to be studied in a tissue-specific context and may vary functionally and structurally from the canonical PGC-1α [29,30,31].

### 2.2. Regulation of the Master Regulator

**Figure 2:**
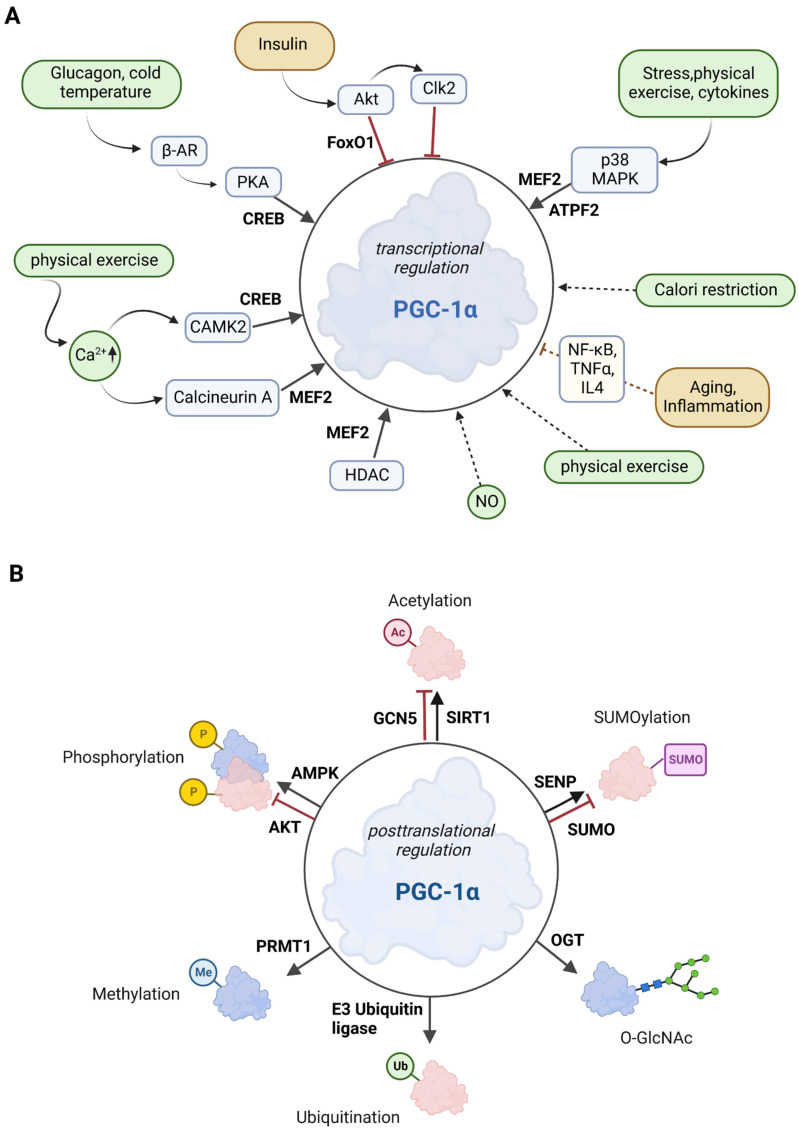
Overview of the effectors of PGC-1α. (**A**) Posttranscriptional control of PGC-1α. The upregulators are in green boxes, while the downregulators are shown in yellow color. Transcription factors that control PGC-1α gene expression are printed in bold. The posttranslational mediators are in light blue. Reprinted/adapted with permission from Ref. [36]. Copyright year 2021, copyright owner’s name Taylor and Francis, Licence No: 5532960029681. Adapted from Hyttinen et al., 2021 [36]. (**B**) Posttranslational modifications of PGC-1α. Several sites for modifications including phosphorylation, acetylation, methylation, ubiquitination, O-GlcNAcylation (O-linked N-acetyl glucosylation), and SUMOylation have been described for PGC-1α. Red molecules indicate inactivation, and blue indicates activation. Adapted from Fernandez-Marcos and Auwerx, 2011 [32]. Created with BioRender.com, agreement No: NJ25C9I2WL.

PGC-1α is tightly regulated at two different levels: Firstly, PGC-1α is regulated at the transcriptional level (see also Section 2.1) through several transcription factors and various extracellular stimuli such as insulin/glucagon levels, Ca^2+^, temperature, or exercise via signaling cascades. Secondly, PGC-1α is regulated at the posttranslational level through numerous modifications, such as acetylation, phosphorylation, methylation, or ubiquitination [32,33]. The overview in Figure 2 shows the most important signaling routes that target PGC-1α through posttranslational and transcriptional modifications.

### 2.3. Stress-Related Transcriptional Regulation of PGC-1α

Various factors play a role in the regulation of PGC-1α, e.g., the transcription of PGC-1α is upregulated by forkhead box class-01 (FoxO1), myocyte enhancer factor 2 (MEF2), activating transcription factor 2 (ATF2), and cyclic AMP response element-binding protein (CREB). These upstream factors are induced by several extracellular stimuli such as stress, exercise, or cytokines [32,34]. Early-phase mediators of inflammation, such as the tumor necrosis factor α (TNFα), interleukin-4, and interferon-γ, regulate PGC-1α gene expression. Nuclear factor-kappa B (NF-κB), mitogen-activated protein kinase (MAPK), or Akt serine/threonine kinase (protein kinase B; Akt; PKB) have a mediating effect on these signaling pathways as well [35]. The relationship between cytokine signaling and PGC-1α in the context of inflammation will be discussed in detail in Section 4.1.

p38 MAPK activates MEF2 and ATF2, both of which stimulate PGC-1α expression. Exercise increases Ca^2+^ levels, leading to the activation of MEF and CREB factors via calcineurin A and Ca^2+^/calmodulin-dependent protein kinase 2 (CAMK2) [32,37]. Activation of 5′ adenosine monophosphate-activated protein kinase (AMPK) via CAMK2 is promoted by calcium ions [38]. FoxO1 and Akt are activated by insulin, whereas glucagon (via glucagon receptor) and cold temperature (via β3-adrenergic receptor; β-AR) stimulate protein kinase A (PKA), which subsequently promotes CREB-mediated transcription [32]. In summary, the combination of a variety of response factors, integrating environmental and intracellular stimuli, controls the PGC-1α gene expression [36].

Class II histone deacetylases (HDACs) inhibit the MEF2 transcription factor and indirectly regulate PGC-1α gene expression. In HDAC-negative mouse models, MEF2 activity was increased, resulting in enhanced skeletal muscle development (endurance and resistance to fatigue) [39]. This observation may be related to an increase in the PGC-1α expression [36].

Recruitment of RNA polymerase II to the PGC-1α promoter is inhibited by its phosphorylation by cyclin-dependent kinase 9 [40]. In addition, transcription factors EB (TFEB)and E3 (Tfe3), have been reported to directly regulate the PGC-1α gene [41].

In addition, various epigenetic modifications of the PGC-1α promoter regulate PGC-1α gene expression [42]. Barrès et al. showed that promoter methylation in muscle cells by DNA methyltransferase 3B (DNMT3B) leads to the repression of the PGC-1α gene in the presence of high levels of FA [43]. This is relevant with respect to the involvement of PGC-1α in the control of mitochondrial biogenesis and the regulation of mitophagy [33,44]. Aging and inflammation, which are often associated with an increase in ROS, also affect PGC-1α gene expression. This will be further discussed later in Section 4.

### 2.4. Posttranslational Regulation of PGC-1α

AMPK, MAPK, Akt, S6 kinase, and glycogen synthase kinase 3β (GSK3β) are the major and best-described protein kinases that target PGC-1α for posttranslational phosphorylation (Figure 2B) [32,45]. Here, we will discuss AMPK and Akt in more detail.

AMPK is activated when the cellular AMP/ATP ratio increases. It is, therefore, a key enzyme in mitochondrial energy homeostasis. Increased AMPK activity results in the inhibition of cell growth, proliferation, and anabolic processes such as lipid synthesis [46]. Specifically, AMPK binds to PGC-1α in muscle cells and phosphorylates Thr177 and Ser538. This phosphorylation increases the transcriptional activity of PGC-1α. Furthermore, these phosphorylations are required for AMPK-induced gene expression of mitochondrial genes, glucose transporter 4 (GLUT4), and PGC-1α itself [47]. In addition, increased protein stability is a result of the p38 MAPK-induced phosphorylation of PGC-1α at Thr262, Ser265, and Thr298 [32]. In conclusion, cellular energy balance is primarily regulated by the AMPK/PGC-1α axis, which largely controls mitochondrial energy metabolism. This balance can be disrupted by chronic overnutrition, which triggers the shutdown of AMPK expression and leads to impaired PGC-1α activity, resulting in mitochondrial dysfunction [15,47].

Akt is involved in several cellular signaling and regulatory pathways, such as PGC-1α regulation. It is known that phosphorylation can also decrease PGC-1α activity in cells; for example, Akt can phosphorylate several C-terminal sites of PGC-1α. By phosphorylation of PGC-1α, Akt inhibits both gluconeogenesis and fatty acid oxidation (FAO) [48]. Akt, activated in the liver upon feeding, phosphorylates PGC-1α at Ser568 and Ser572, which inhibits the gluconeogenic program of downstream targets. However, these specific phosphorylations do not affect the function of PGC-1α as an activator of mitochondrial and FAO genes [49]. Akt is also involved in the phosphate-3-kinase-Akt-mechanistic target of rapamycin (mechanistic target of rapamycin; mTOR) signaling pathway. This pathway controls several cellular mechanisms (survival, differentiation, growth, metabolism, and cancer) and inhibits the PGC-1α response [50,51]. In addition, Akt activates CDC2-like kinase 2 (Clk2), which also mediates the PGC-1α inactivation [52]. Akt also inhibits the PGC-1α-mediated activation of the FoxO1 transcription factor [32,53]. In 3T3 cells, the stability of PGC-1α is regulated by GSK3β, which targets PGC-1α for intranuclear proteasomal degradation [54].

Sirtuin 1 (SIRT1) belongs to the family of the silent information regulator 2-related histone deacetylase family [55]. To mediate the deacetylation of target substrates, sirtuin proteins require nicotinamide adenine dinucleotide (NAD) [56]. Since the cellular REDOX balance of NAD^+^ and NADH is closely linked to catabolic metabolism, it is proposed that SIRT1 acts as a sensor that directly links metabolic perturbations to transcriptional outputs. As such, SIRT1 interacts with PGC-1α and deacetylates it in an NAD^+^-dependent manner (Figure 2B) [57]. It has been suggested that PGC-1α and SIRT1 are mitochondrially imported proteins localized in the mitochondrial matrix [58], but the evidence suggest that SIRT1 is a nuclear/cytosolic protein [59], while SIRT3is located in the mitochondria [60]. SIRTs primarily affect mitochondrial function, with two existing pathways: a PGC-1α-dependent and a PGC-1α-independent pathway [15]. In the PGC-1α-dependent pathway, SIRT1 activates PGC-1α through deacetylation. Activated PGC-1α acts as a coactivator for mitochondrial transcription factor A (TFAM), which is thought to promote the transport of SIRT1 and PGC-1α into mitochondria where they form a complex with the D-loop region of mtDNA. The D-loop region regulates mitochondrial DNA replication and transcription [58,59]. SIRT1 activity can be enhanced by exercise and fasting [61,62]. Fasting has been shown to induce SIRT1-dependent PGC-1α deacetylation in skeletal muscle and is required for the activation of mitochondrial FAO proteins under low glucose conditions [63]. In contrast, histone acetyltransferase activity controls non-depressible 5 (GCN5), which results in PGC-1α acetylation. In addition, the SIRT1 inhibitor, nicotinamide, induces PGC-1α acetylation, thereby reducing the expression of PGC-1α target genes. Cellular energy overload leads to increased levels of steroid receptor coactivator 3 (SRC-3), resulting in GCN5 up-regulation and thus pronounced PGC-1α acetylation [55].

Specific ubiquitination (Ub) of PGC-1α by the E3 ubiquitin ligase SCF^Cdc4^ (Skp1/Cullin/F-box cell division control 4) results in a very short half-life (0.3 h) of PGC-1α in the nucleus due to proteolytic digestion (Figure 2B) [64]. Conversely, decreased SCF^Cdc4^ activity results in PGC-1α accumulation in response to oxidative stress, thus providing an increased ability to neutralize toxic metabolic byproducts such as ROS [65].

The small ubiquitin-like modifier (SUMO)-1 protein attenuates the activity of PGC-1α through SUMOylation [66]. SUMOylation of PGC-1α inactivates the enzyme, which is reversed by a Sentrin/SUMO-specific protease (SENP1) that de-SUMOylates PGC-1α and thus results in mitochondrial biogenesis [67].

Methylation by the protein arginine methyltransferase 1 (PRMT1) increases PGC-1α activity and induces the transcription of genes important for mitochondrial biogenesis [68] (Figure 2B).

Another posttranslational modification is O-GlcNAcylation, which is the addition of β-N-acetylglucosamine (GlcNAc) groups by O-linked β-N-acetylglucosamine (O-GlcNAc) transferase (OGT). This stabilizes PGC-1α by inhibiting its ubiquitination [69] (Figure 2B). In addition, O-GlcNAcylation of the transcription factor FoxO1 and the CREB-regulated transcription co-activator 2 (CRTC2) is associated with PGC-1α activity. During its interaction with PGC-1α, OGT transfers a GlcNAc group to FoxO1 and is then able to modify CRTC2. This is thought to be necessary for the interaction of CRTC2 with PGC-1α, resulting in increased PGC-1α gene expression. O-GlcNAcylation of specific transcription-related factors, such as PGC-1α and FoxO1, is important for nutrient stress sensing and cellular energy metabolism [70].

In summary, various posttranslational modifications create a versatile and efficient array for regulating the activity and intracellular localization of PGC-1α, thus ultimately contributing to the pivotal role of PGC-1α in mitochondrial energy metabolism and biogenesis [71].

## 3. The Link between PGC-1α and Mitochondria

### 3.1. PGC-1α as the Master Regulator of Mitochondrial Biogenesis

**Figure 3:**
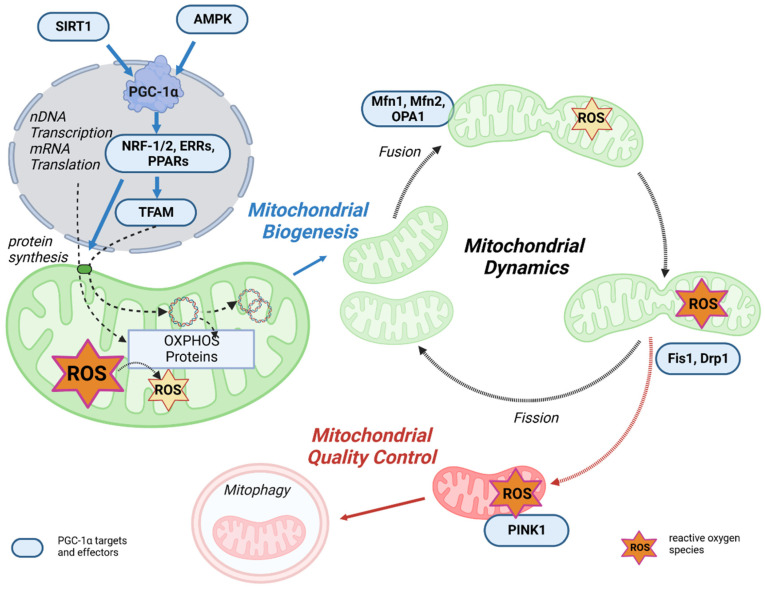
The link between mitochondrial life cycle and PGC-1α. Mitochondrial biogenesis is initiated by an energetic imbalance sensed by two pathways: AMPK and SIRT1. Increased expression or activity of the key regulator of mitochondrial biogenesis PGC-1α activates the expression of NRF-1/2, which induces the expression of TFAM, which translocates to mitochondria, binds to mtDNA, and activates transcription and replication. An increase in OXPHOS proteins reduces ROS generation in mitochondria. Mitochondrial fusion and fission dynamics are also affected by ROS. Dysfunctional mitochondria can be eliminated through a process known as mitophagy. Adapted with permission from Ref. [79], Copyright year 2017, copyright Portland Press LTD. Created with BioRender.com, agreement No: PV25C9IVVA.

PGC-1α is the master regulator of mitochondrial biogenesis and an important regulator of mitochondrial oxidative capacity (Figure 3). This occurs through a variety of transcription factors, such as ERR, PPARγ, and NRF-1/2, which are coactivated by PGC-1α and all play an important role in mitochondrial oxidative capacity [72,73]. In addition, the interaction between PPARγ and PGC-1α can stimulate mitochondrial biogenesis through the regulation of PGC-1α activity itself. Specifically, PGC-1α and PPARγ control proteins involved in the regulation of mitochondrial biogenesis, including promoting OXPHOS gene expression in the nucleus and mitochondria and stimulating mtDNA replication, thereby enhancing mitochondrial function and metabolism [74,75,76]. The upstream gatekeepers of PGC-1α activity are SIRT1 and AMPK, which are important actuators in the regulatory network of metabolic homeostasis [77,78].

Mitochondrial transcription is activated by PGC-1α, PGC-1β, and PRC, but PGC-1α is the master regulator of mitochondrial biogenesis. The process is initiated when PGC-1α is activated by phosphorylation of AMPK or deacetylation of SIRT1 and stimulates various nuclear transcription factors, such as NRF-1, NRF-2, and estrogen-related receptor alpha (ERRα). Through activation of NRF-1/2 [80], PGC-1α promotes TFAM transcription and expression [81,82]. In addition, NRF-2 regulates the gene expression of the protein import receptor Tom70 (Tom70) of the translocase of the outer mitochondrial membrane (TOM) [83]. TFAM stimulates the transcription and replication of mtDNA [84,85], but the correlation between TFAM levels and mtDNA transcription and replication may be complex [86]. In the next step, specific translation factors, such as mtIF2 and mtIF3, translate the mtDNA. In terms of energy metabolism, the PGC-1α-NRF-1/2 pathway promotes the gene expression of mitochondrial complexes I, II, III, IV, and cytochrome *c,* thereby activating OXPHOS [87].

In summary, mitochondrial biogenesis must undergo mtDNA transcription and translation, demonstrating that upregulation of transcription factor activation via PGC-1α is a key step in mitochondrial biogenesis.

### 3.2. PGC-1α Affects Mitochondrial Dynamics and Quality Control

Complementary to mitochondrial biogenesis, mitochondrial quality control is a key process for maintaining the energy supply by mitochondria. Mitochondrial quality control is a multilevel process involving multiple mitochondrial and cytosolic proteases, protein replenishment, and mitophagy [88]. Maintenance of mitochondrial performance and adaptation to changing energy demands is regulated by remodeling mitochondrial structures, which is primarily controlled by fission/fusion, mitochondrial biogenesis, and mitophagy [89]. Intriguingly, in addition to its role in regulating mitochondrial biogenesis, PGC-1α is also involved in mitochondrial dynamics and mitophagy [90]. An overview of these processes is shown in Figure 3.

It is well known that mitochondria are dynamic organelles that continuously undergo the processes of fusion and fission. Mitochondrial fusion is controlled by mitofusin 1 (Mfn1) and mitofusin 2 (Mfn2) in the mitochondrial outer membrane and optic atrophy 1 (Opa1) in the mitochondrial inner membrane [91,92]. These fusion proteins contain functional GTPase domains and, upon activation, result in an expanded, branched mitochondrial network. Mitochondrial fission is the process that counteracts fusion and allows the mitochondrial network to split, resulting in small, fragmented, and globular mitochondria. Fission is also regulated by GTPase proteins, such as fission 1 protein (Fis1) and dynamin-related protein (Drp1) [93,94]. Healthy mitochondrial dynamics are regulated by maintaining a balance between these opposing processes, which is fundamental to maintaining mitochondrial quality and function [89]. According to Peng et al., it can be speculated that excessive mitochondrial fragmentations lead to mitochondrial dysfunction [95]. PGC-1α can slow down the fission of neuronal mitochondria by regulating Drp1 levels [96], resulting in increased mitochondrial fusion. This may prevent or slow down the damage and denaturation of neuronal axons caused by ATP depletion induced by mitochondrial fragmentation. Activation of PPARγ decreases Drp1 activity through phosphorylation, thereby reducing excessive mitochondrial fission and neuronal damage [97]. The beneficial effect of PPARγ/PGC-1α to reduce mitochondrial oxidative stress response via stimulation of mitochondrial biogenesis, dynamics, and function was also demonstrated in a rabbit model of diabetes associated with atrial ROS stress [98]. PGC-1α was shown to directly regulate Mfn1 gene transcription by coactivating the ERRα, which ultimately promotes mitochondrial fusion (Figure 3) [99]. In PGC-1α overexpression and knock-out cell models, PGC-1α was shown to regulate Mfn2 and p-Drp1 protein expression and phosphorylation [95], which are important for the balance between mitochondrial dynamics, function, and homeostasis. In summary, PGC-1α provides a link between mitochondrial biogenesis and fission/fusion [95].

Finally, mitochondrial degradation by autophagy is also mediated by PGC-1α through transcriptional mechanisms [100]. Autophagy is the major process by which damaged organelles and cellular by-products are degraded and recycled in the lysosome to maintain cellular homeostasis [101]. Mitophagy is the mitochondria-specific form of autophagy. Damaged and/or dysfunctional mitochondria are often characterized by a disturbance of the mitochondrial membrane potential, which may also have effects on the sensitive ROS balance [102]. Depolarization of mitochondria leads to the recruitment of PTEN-induced kinase 1 (PINK1), which activates parkin, an E3 ubiquitin ligase, followed by ubiquitination of outer membrane proteins. This mitochondria-ubiquitinated complex is then engulfed by the autophagosome and degraded in the lysosome. Mitophagy is critical for maintaining healthy mitochondria in various tissues and disease states by deleting defective mitochondrial segments within the network [89]. In addition, PGC-1α can interact with and stabilize the mRNA of mitostatin, a mitochondrial protein associated with oncostatic (=Tumor inhibiting) activity. This induces mitostatin-dependent mitophagy, which leads to negative feedback regulation of vascular endothelial growth factor A (VEGF-A) production, thereby attenuating tumor angiogenesis.

Although PGC-1α expression generally counteracts the process of autophagy, it can promote mitophagy to maintain mitochondrial homeostasis in this specific scenario, further illustrating the complexity of the role of PGC-1α in regulating autophagic signaling pathways [71]. Mitochondrial biogenesis and mitophagy are tightly coupled, and a balanced interplay between these two processes is critical for cellular adaptation and stress resistance [103]. PGC-1α is a key player in these processes.

## 4. PGC-1α, Mitochondria, and Oxidative Stress

**Figure 4:**
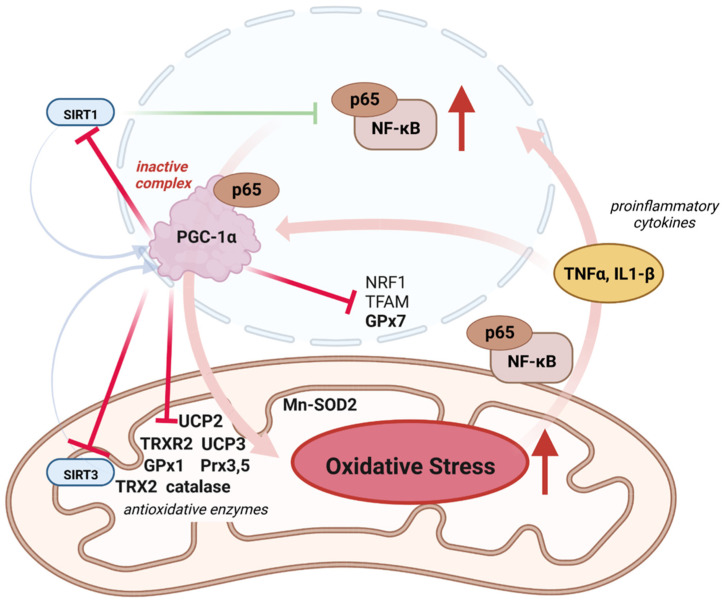
Mitochondrial ROS defense is impaired by PGC-1α downregulation under inflammatory conditions. NF-κB decreases PGC-1α expression by modulating proinflammatory cytokines, and its subunit p63 decreases PGC-1α activity by forming a complex. The reduced PGC-1α activity results in the downregulation of its antioxidant target genes and ultimately results in increased oxidative stress. Low PGC-1α levels and concomitant oxidative stress further promote NF-κB activity, thereby exacerbating the inflammatory response. Mitochondria accumulate adjacent to the nucleus [122]. Created with BioRender.com, agreement No: AW25C9KBZ5.

PGC-1α levels control the expression of OXPHOS genes and oxidative stress response genes in human, mouse, and bovine endothelial cells (Figure 4) [104]. PGC-1α itself is controlled by SIRT3, a mitochondrial deacetylase [105]. SIRT3 likely influences glucose metabolism by inducing an anti-Warburg effect [106], and thus indirectly reduces ROS levels. Through the SIRT3/PGC-1α axis, ROS levels are further reduced due to an increased expression of the antioxidant enzymes, including Mn-dependent superoxide dismutase (MnSOD), catalase, mitochondria-localized peroxidases Prx3 and Prx5, and the oxidoreductases thioredoxin TRX2 and TRXR2, which are the members of the conserved superfamily of Thioredoxin (TRX enzymes that function as cysteine reductases, as well as the uncoupling protein UCP-1 [104]. The members of the peroxidase class III family de-toxify peroxide with thioredoxin and/or glutathione as electron donors. Glutathione peroxidase (GPx) and peroxiredoxin have protective effects against ROS in spermatozoa during post-testicular maturation [107]. Uncoupling proteins (UCPs) increase the leak of the inner mitochondrial membrane for protons and thus decrease the proton motive force Δ*p*. Since ROS production and Δ*p* are positively correlated, uncoupling via UCPs protects against oxidative damage, as shown, for example, for UCP-2 and UCP-3 as UCP-1 homologs in the heart [108]. Thus, UCPs, in particular UCSP-2, are key regulators of metabolism and mitochondrial ROS [109].

### 4.1. Inflammation, ROS, and PGC-1α

Oxidative stress in mitochondria is induced when the inflammation-related transcription factor NF-κB is activated, e.g., by cytokine signaling (Figure 4). Under normal conditions, PGC-1α regulates levels of pro-inflammatory cytokine levels through its physical interaction with the NF-κB subunit p65 (p65). More specifically, PGC-1α blocks NF-κB transcriptional activity toward its target genes, including those encoding pro-inflammatory cytokines [110,111,112]. Certain cytokines, such as TNFα and interleukin 1β (IL1-β), increase NF-κB/P65 levels. In human and murine cardiac cells, the NF-κB subunit p65 was found to interact with PGC-1α, thereby inactivating PGC-1α. Notably, this interaction is stabilized upon TNFα-dependent NF-κB activation [112]. Thus, the PGC-1α—NF-κB/P65 interaction is an important hub in inflammatory pathways. When PGC-1α activity is decreased under inflammatory conditions due to high cytokine levels, this further enhances the inflammatory response [113,114,115], which is exacerbated by increased oxidative stress, among other factors. This is due in part to the TNFα-induced decrease in gene expression of ROS-scavenging enzymes in the cytosol and mitochondria. For example, the antioxidant enzyme superoxide dismutase (SOD1), which has ROS-scavenging activity but is also involved in the activation of nuclear gene transcription or as an RNA binding protein, is decreased [116]. In addition, the oxidative stress sensor/transmitter GPx7, which has multiple roles in redox homeostasis, is affected. The deficiency of GPx7 in mice or humans is associated with ROS accumulation [117]. The increased physical interaction between p65 and PGC-1α after TNFα-induced NF-kB activation is responsible for the reduction of PGC-1α activity and expression [112], a metabolic switch towards glycolysis, and subsequent dysregulation of the mitochondrial antioxidant defense [104]. Similarly, PGC-1α levels are downregulated in C2C12 cells after TNFα and IL1-β treatment [118,119] or in cardiac cells after lipopolysaccharide (LPS) and TNFα exposure [120]. Remarkably, the regulation of PGC-1α levels by LPS appears to be tissue-specific. In skeletal muscle, LPS induces PGC-1α expression after short-term exposure but decreases its transcript levels 24 h after LPS injection. Interestingly, LPS decreases hepatic PGC-1α levels early on, but its expression levels recover 8–16 h after injection [121].

Figure 4 summarizes the role of PGC-1α in anti-inflammatory defense and shows how the PGC-1α/p65 complex leads to increased oxidative stress by impairing the detoxification of ROS, mainly by reducing gene expression of key antioxidants [123]. Low levels of PGC-1α or decreased activity of PGC-1α in inflamed tissues increase ROS generation and cause oxidative damage [110,124,125,126]. In this context, it is interesting to note that PGC-1α is involved in the regulation of macrophage polarization from the pro-inflammatory M1 to the anti-inflammatory M2 type. Altered PGC-1α levels disturb the balance of macrophage types during inflammation. After LPS/TNFα injection, PGC-1α overexpression in skeletal muscle does not suppress pro-inflammatory cytokine expression but decreases M1 cytokine IL-12 levels and enhances the generation of anti-inflammatory M2 cytokines [115]. In line with this, experiments with PGC-1α negative mice showed increased M1 and decreased M2 responses in kidney cells after AKI induction [127,128].

In conclusion, PGC-1α and NF-κB are mutually regulated during inflammation, in which oxidative stress plays an essential role. During inflammation, NF-κB downregulates the expression and stability of PGC-1α. Low PGC-1α activity reduces the translation of its antioxidant target genes, resulting in oxidative stress. On the other hand, low PGC-1α levels and concomitant oxidative stress promote NF-κB activity, thereby exacerbating the inflammatory response [42].

### 4.2. Role of PGC-1α in Mitonuclear Crosstalk for ROS Defense

**Figure 5:**
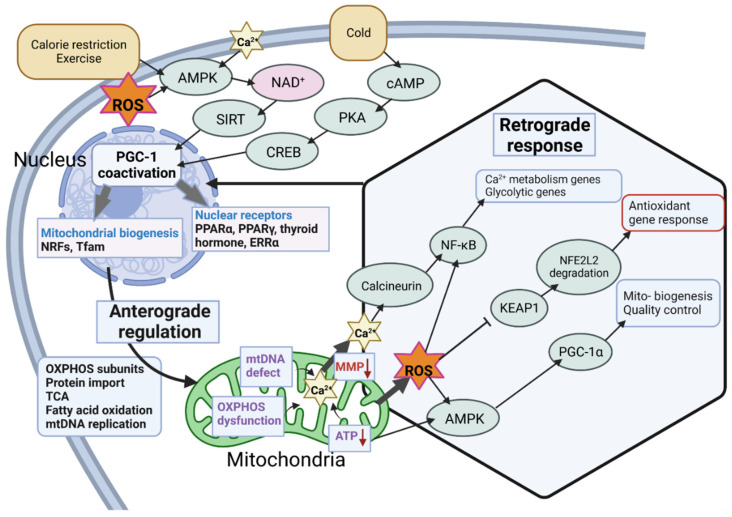
PGC-1α is a key regulator of ROS defense involving mitonuclear communication. Anterograde regulation results in the biogenesis of OXPHOS and other mitochondrial pathways, while perturbations in mitochondria initiate retrograde communications signals to the nucleus to recalibrate quality control, antioxidant response, and Ca^2+^ metabolism. See main text for details. Created with BioRender.com, agreement No: HD25C9KU1V.

The internal accumulation of ROS in the cytosol and mitochondria poses a threat to cells when certain thresholds are crossed (Figure 5). To modulate the response to ROS, the nucleus and mitochondria communicate via anterograde and retrograde signaling. In general, mitonuclear crosstalk is critical for meeting metabolic demands [129]. Activation of nuclear-mediated anterograde signaling by elevated ROS levels, for example, is mediated by PGC-1α. The primary role of PGC1α as a transcriptional regulator has numerous downstream consequences and feedback mechanisms, as demonstrated in Figure 5. Specifically, under conditions of oxidative stress, PGC1α is required for antioxidant defense by inducing the expression of ROS-detoxifying proteins, such as GPx1 and SOD2 [130]. These proteins play a critical role in preventing cell death [131,132]. An imbalance in PGC-1α disturbs the redox/ROS balance in cells, ultimately affecting their inflammatory response (Figure 4 and Figure 5). However, under certain conditions, PGC1α activity increases following increased ROS production after an inflammatory stimulus resulting in PGC1α-mediated gene activation [133]. Then, PGC-1α controls the mitochondrial response by regulating OXPHOS gene expression (Figure 5). Two nuclear transcription factors, NRF-1 and GA-binding protein-α (GABPα also NRF2α), are primarily accountable for this response [134]. For example, NRF-1 regulates cytochrome *c* gene expression and directly or indirectly modulates the levels of the OXPHOS proteins [134], which increases the capacity of the electron transport chain and prevents the over-reduction of the CoQ pool. Over-reduction of the CoQ pool is a threat because it increases the likelihood of reverse electron flow (RET) and increased ROS production at complex I [135]. The role of cytochrome *c* in ROS production depends on its phosphorylation status [136]. Increased OXPHOS activity would also decrease the NADH/NAD^+^ ratio, which is a determinant of ROS production, at least in isolated mitochondria [137,138]. Thus, there is accumulating evidence that increasing OXPHOS capacity (which is not equal to maximal respiration) has beneficial effects on reducing ROS production. However, exact relationships are difficult to unravel because ROS are generated at multiple sites and are diverse, and ROS detection is also challenging.

The molecular mechanism is as follows: NRF-1 binds to the promoters of the mitochondrial replication factors TFAM [139], TFB1M, and TFB2M [140], activating their expression and thus mtDNA replication. TFAM is a highly abundant mitochondrial protein and a key factor for mitochondrial transcription initiation. Using super-resolution microscopy, molecular modeling, and volume calculations, TFAM was shown to be the major constituent of mitochondrial nucleoids, protein-mtDNA complexes that contain approximately 1,000 TFAM molecules per mtDNA molecule in human cultured cells [141]. TFAM binds specifically to both mtDNA promotors recruiting RNA polymerase (POLRMT) and mitochondrial transcription factor B2 (TFB2M). In addition, TFAM binds mtDNA in a sequence-unspecific manner, thereby enhancing mitochondrial DNA packaging by promoting looping [142]. Homozygous disruption of *Tfam* in chicken DT40 cells was lethal, whereas *tfam*^+^*/tfam*^–^ heterozygotes had a 40–60% reduction in mtDNA and mtDNA transcription [143]. Using inducible tetracycline-regulated overexpression of TFAM in human HeLa cells, a direct correlation between TFAM expression and mtDNA content was found; however, TFAM expression levels were not correlated with mtDNA transcription [144]. It appears that TFAM expression directly modulates the maintenance of mtDNA copy number, which is essential for normal cellular function. Up to thousands of mitochondria are present in mammalian cells. The number is variable, depending on the cell type and its energetic requirements. For example, mammalian hepatocytes contain approximately 800 mitochondria per cell, whereas mammalian oocytes are estimated to have more than 100,000 [145].

In addition to PGC-1α, PPARs (PPARγ, PPARα, and PPARβ) are involved in the mitonuclear stress response. These three nuclear receptor isoforms regulate many biological processes by binding to PPAR-specific response elements (PPREs) within the promoter region, thereby unwinding the chromatin structure, and initiating the transcription of specific genes (Figure 4 and Figure 5) [146]. PPARs are involved in FA transport, β-oxidation, activation of the synthesis of many FAs and FA derivatives, and mitochondrial respiration [147]. PPARs play critical roles as lipid sensors and modulators of lipid metabolism and are involved in a wide range of biological processes, including inflammation, cellular growth, cancer development, apoptosis, and differentiation [148]. It should be noted that the transcriptional control of mitochondrial biogenesis is tissue- and organ-specific, and different stimuli can trigger different downstream pathways [149]. This will be discussed in the next section.

### 4.3. PGC-1α, ROS, and Disease

Over the past few decades, human lifestyles and, especially, diets have changed. Being overweight and obese is the result, leading to diseases such as type 2 diabetes, fatty liver disease, and cardiac disease. Such diseases are summarized under the term metabolic diseases and are associated with high blood glucose levels, high blood pressure, high blood lipid levels, and high cholesterol levels [150], often related to increased ROS levels. In this context, PGC-1α dysregulation may alter the metabolic properties of tissues and thus be involved in various metabolic disorders. Indeed, single nucleotide polymorphisms in the human PGC-1α gene are associated with obesity, diabetes, and hypertension [151]. Energy starvation and/or reduced catabolic rate can be detected by AMPK and SIRT1, whose activation increases PGC-1α-dependent transcription. During high-calorie diets or situations where energy is not limited, AMPK activity is turned off by the high levels of intracellular ATP. Similarly, high-fat diets increase SRC-3, which positively regulates protein levels of the acetyltransferase GCN5, which in turn plays the opposite role of SIRT1 in PGC-1α acetylation, thereby decreasing PGC-1α transcriptional activity. Disruptions in this metabolic network that controls PGC-1α activity can significantly contribute to systemic metabolic complications [55].

Aging represents a progressive disruption of the homeostasis of physiological systems. It leads to structural destruction, organ dysfunction, and increased susceptibility to injury and disease [152]. With continued cell division, telomeres gradually shorten and eventually enter a dysfunctional state, leading to cell growth arrest and senescence. The deficiency of telomerase reserve transcriptase (TERT) in mice leads to telomere dysfunction and shortening with DNA damage [153]. Studies using TERT-knockout mice showed decreased PGC-1α gene expression [153,154]. While PGC-1α deletion leads to cellular senescence characterized by telomere shortening, DNA damage, and increased p53 levels, ectopic expression of PGC-1α has beneficial effects on telomere function [155]. During telomere dysfunction, p53 can be activated to bind and repress PGC-1α promoters. This repression leads to mitochondrial dysfunction in the liver and heart in age-related dilated cardiomyopathy, defects in hepatic gluconeogenesis, and reduced reconstitution capacity of hematopoietic stem cells [153]. Accumulating evidence suggest an anti-aging effect of PGC-1α in various organs, but more research is needed to identify clear PGC-1α-related targets for treatment [152]. Studies using muscle-specific PGC-1α knockout mice have shown that a decrease in PGC-1α and reduced mitochondrial oxidative capacity potentiate the development of glucose intolerance and insulin resistance associated with aging [156]. Paradoxically, another study showed that PGC-1α overexpression in muscle leads to insulin resistance in young mice fed a high-fat diet [157].

Furthermore, PGC-1α may play a role in the response of cancer cells to the environment. First, the supply of nutrients and oxygen in tumors fluctuates, forcing cancer cells to adapt their metabolism, relying alternatively on glycolysis or OXPHOS. Such fluctuations alter the energy status of cancer cells and interfere with signaling pathways (AMPK, mTOR), transcription factors (hypoxia-inducible factor 1 alpha; HIF1α), and proteins (glucose transporter; GLUT) known to be associated with PGC-1α activity [11]. Second, common cancer treatments such as radiation or chemotherapy have been shown to induce oxidative stress in tumor cells [158,159]. ROS overproduction can either induce cell death or resistance to treatment through mechanisms involving antioxidant enzymes [160]. ROS are mainly produced by mitochondria, and thus regulation of mitochondrial biogenesis and PGC-1α may interfere with treatment response [11]. Finally, studies have shown that lipids are an energy source for cancer cells. Adipocytes are important members of the tumor microenvironment. They promote cancer cell aggressiveness by releasing cytokines (adipokines), such as interleukin 6 to increase the invasive properties of breast cancer cells, or chemokine (C-C motif) ligand 7, resulting in the local proliferation of prostate cancer cells [161,162]. In addition, adipocytes release FA from lipid droplets that are oxidized by fatty acid β-oxidation in cancer cells. This metabolic interaction between adipocytes and cancer cells promotes aggressiveness and metastasis [163]. Although the majority of the literature supports the tumorigenic activity of PGC-1α, there are also paradoxical antineoplastic effects in some tumor types [164,165,166,167,168]. In renal cell carcinoma, increased mitochondrial activity is induced by PGC-1α. This activity is closely associated with increased ROS production, leading to increased oxidative stress. Thus, ectopic expression of PGC-1α leads to impaired tumor growth and increased sensitivity to cytotoxic therapies [165].

### 4.4. PGC-1α as a Drug Target

In the last decade, increased PGC-1α activity as a potential therapeutic target has come into focus, especially in aging research [36,152,169]. Therefore, it is important to know which and how drugs increase PGC-1α activity in order to assess their potential for potential treatments.

Resveratrol is a natural polyphenolic phytochemical with anti-inflammatory, antioxidant, anti-diabetic, and neuroprotective effects [170]. The SIRT1 pathway activated by resveratrol is associated with deacetylating activity, leading to changes in several downstream regulators such as PGC-1α [171]. Increased SIRT1 activity, triggered by elevated NAD^+^ levels, upregulates PGC-1α transcriptional activity [57]. However, resveratrol does not bind to the native peptide of SIRT1 or full-length protein substrates. Regardless of the direct target of resveratrol, SIRT1 remains one of the most intensively studied targets associated with the anti-aging effects of resveratrol [170]. Niu et al. showed in recent studies that dietary resveratrol activates the AMPK/PGC-1α axis, thereby promoting the biogenesis of obesity-damaged mitochondria and muscle regeneration [172].

Metformin is a synthetic antidiabetic drug that activates PGC-1α via AMPK [173] and also downregulates mTOR and ROS production [174]. Metformin has multiple targets, including direct or indirect (through AMPK signaling) inhibition of complex I [175,176,177]. Recent research has also shown that metformin and other biguanides can directly inhibit cytochrome c oxidase [178]. However, it is important to note that the effects of metformin are strongly dose-dependent. Complex I inhibition is only observed at metformin concentrations higher than those typically achieved in vivo (>1 mM) [179,180,181]. An alternative mechanism of metformin action has been proposed [178,182,183], suggesting that alterations in the hepatic redox state and inhibition of glycerol-3-phosphate dehydrogenase (GPD2) potentiate its glucose-lowering effects. This alternative mechanism is significant because it occurs at lower concentrations of metformin. The described anti-aging effects of metformin are based on reduced insulin levels and a consequent reduction in insulin-like growth factor 1 (IGF1) signaling and glucose levels [184].

Rapamycin, a macrolide immunosuppressant, works primarily by inhibiting mTOR. Studies have shown that rapamycin extends the lifespan of mice [185,186,187,188,189,190,191]. Inhibition of mTOR is one of the major effects of AMPK. Rapamycin can, therefore, phenocopy some effects of AMPK and also ameliorate renal fibrosis by blocking mTOR signaling in interstitial macrophages and myofibroblasts [192]. However, mTOR can activate Yin Yang 1 (YY1), a transcription factor that increases the activity of the PGC-1α promoter [193,194].

Fenofibrate is a drug for hypertriglyceridemia and mixed dyslipidemia. Fenofibrate has lipid-modifying effects through the activation of PPARα and also protects against age-related changes in the kidneys. It increases the phosphorylation of AMPK and the activation of PGC-1α [195].

Bezafibrate, a drug used to treat hyperlipidemia, activates the PGC-1α/PPAR pathway and increases mitochondrial biogenesis and fatty acid β-oxidation in mice. In humans, bezafibrate is believed to exert effects in tissues with chronic bioenergetic degenerative conditions [196]. A recent research article demonstrated that Bezafibrate is a potential pharmacological candidate for disorders with MGA (3-Methylglutarate) accumulation, such as Barth syndrome and dilated cardiomyopathy with ataxia syndrome. MGA had a strong tendency to reduce PGC-1α activity. Pretreatment with bezafibrate prevents MGA-induced oxidative stress and mitochondrial dysfunction [197].

Rosiglitazone is a PPARγ agonist, which is used as an antidiabetic agent. It has also been reported to increase the nuclear fraction of PGC-1α in a mouse model of renal fibrosis, exert protective effects against oxidative stress, and reduce epithelial-mesenchymal transition (EMT)-derived fibrosis [198].

Adiponectin is a cardioprotective agent in diabetes [199]. Previous studies reported that hypoadiponectinemia impaired AMPK-PGC-1α signaling in diabetic hearts [200]. In a cardiomyocyte model for type 2 diabetes (cells grown in high glucose/high-fat medium), adiponectin partially rescues mitochondrial biogenesis via PGC-1α mediated signaling [199].

In addition to drugs that promote PGC-1α/TFAM activation, there are also agents and substances that downregulate the expression or activation of the mitochondrial biogenesis-associated transcription factors PGC-1α and TFAM and the regulating AMPK [201].

miR-130b-3p is a small non-coding RNA (microRNA; miRNA) that negatively regulates PGC-1α/TFAM biogenesis pathway [202].

2-Methoxyestradiol is a potent anticancer agent that promotes the mitochondrial biogenesis of osteosarcoma cells. 2-Methoxyestradiol affects and downregulates SIRT3 and PGC-1α activity in a concentration-dependent manner, i.e., especially at low physiological concentrations [203].

High-glucose/high-fat conditions: Previous studies showed that mitochondrial biogenesis and function are significantly reduced in adipose and muscle tissue of obese animals [204,205]. High-glucose/high-fat conditions resulted also in altered mitochondrial structure and decreased PGC-1α activity in cardiac myocytes [199].

Cyclosporine A is a drug, used to treat many autoimmune diseases. Qi et al. found that cyclosporine A decreased the expression of PGC-1α at both the mRNA and protein levels in HepG2 cells and inhibited mitochondrial biogenesis [206].

The above-mentioned basic findings can be used as a basis for future clinical approaches in PGC-1α related diseases, such as cancer, metabolic diseases, neurodegeneration, or age-related diseases.

## 5. Conclusions

As shown in this review, PGC-1α is a master regulator of almost all steps of the mitochondrial life cycle: fusion/fission, mitochondrial biogenesis, and mitophagy. It also plays an essential role in mitochondrial redox biology and ROS homeostasis by controlling the expression of ROS-scavenging enzymes on the site and OXPHOS complexes on the other. Through its regulation by sirtuins, NAD^+^-dependent deacetylases, and AMPK, the cell’s energy sensor, PGC-1α also responds to the redox and energy status of the cell, both of which are strongly determined by mitochondria. PGC-1α is thus a key player in the intracellular communication between the cytosol, nucleus, and mitochondria that serves ROS homeostasis. Many known drugs that indirectly or directly improve cellular functions act on mitochondria via PGC-1α as a final target.

## Abbreviations

AMPK	5′ adenosine monophosphate-activated protein kinase
AP	alternative promoter
ATF2	activating transcription factor 2
BAT	brown adipose tissue
CAMK2	Ca^2+^/calmodulin-depend protein kinase 2
Clk2	CDC2-like kinase 2
CREB	cyclic AMP response element-binding protein
CRTC2	CREB-regulated transcription co-activator 2
DNMT3B	DNA methyltransferase 3B
Drp1	dynamin-related protein 1
ER	estrogen receptor
ERRα	estrogen-related receptor α
FA	fatty acid
FAO	fatty acid oxidation
Fis1	fission 1 protein
FoxO1	forkhead box class-01
GABPα	GA-binding protein-α
GCN5	acetyltransferase protein acetyltransferase general control non-depressible 5
GlcNAc	β-N-acetylglucosamine
GPx GPx1 GPx7	glutathione peroxidase glutathione peroxidase 1 glutathione peroxidase 7
GSK3β	glycogen synthase kinase 3β
HDACs	class II histone deacetylases
IGF1	insulin-like growth factor 1
IL1-β	interleukin 1β
LPS	lipopolysaccharide
MAPK	mitogen-activated protein kinase
MEF2	myocyte enhancer factor 2
Mfn1	mitofusin 1
Mfn2	mitofusin 2
MGA	3-Methylglutarate
miRNA	microRNA
MnSOD	Mn-dependent superoxide dismutase
mRNA	messenger RNA
mTOR	mechanistic target of rapamycin
NAD	nicotinamide adenine dinucleotide
NF-κB	nuclear factor-kappa B
NR	nuclear receptor
NRF-1	nuclear respiratory factor 1
NRF-2	nuclear respiratory factor 2
O-GlcNAc	O-linked β-N-acetylglucosamine
OGT	O-linked β-N-acetylglucosamine transferase
Opa1	optic atrophy 1
OXPHOS	oxidative phosphorylation
p65	NF-κB subunit p65
PGC-1α	peroxisome proliferator-activated receptor γ coactivator 1α
PGC-1β	peroxisome proliferator-activated receptor γ coactivator 1β
PKA	protein kinase A
PKB/Akt	protein kinase B
POLRMT	mtDNA promotors recruiting RNA polymerase
PPARs	receptor-like peroxisome proliferator-activated receptors
PPARα	peroxisome proliferator-activated receptor α
PPARγ	peroxisome proliferator-activated receptor γ
PPREs	PPAR-specific response elements
PRC	PGC-1 related coactivator
PRMT1	protein arginine methyltransferase 1
Prx3	peroxiredoxin 3
Prx5	peroxiredoxin 5
ROS	reactive oxygen species
RRM	RNA recognition motif
RS	short serine/arginine-rich stretches
SCF^Cdc4^	Skp1/Cullin/F-box cell division control 4
SENP1	Sentrin/SUMO-specific protease
SIRT1	sirtuin 1
SIRT3	sirtuin 3
SRC-3	steroid receptor coactivator 3
SUMO	small ubiquitin-like modifier
TERT	telomerase reserve transcriptase
TFAM	mitochondrial transcription factor A
TFB2M	mitochondrial transcription factor B2
Tfe3	transcription factor E3
TFEB	transcription factor EB
TNFα	tumor necrosis factor α
Tom70	translocase of outer mitochondrial membrane receptor subunit 70
TRX2	thioredoxin 2
TRXR2	thioredoxin reductase 2
TSS	transcription start site
Ub	specific ubiquitination
UCP-1 UCP-2 UCP-3	uncoupling protein 1 uncoupling protein 2 uncoupling protein 3
VEGFA	vascular endothelial growth factor A
YY1	yin yang 1
β-AR	β3-adrenergic receptor

## References

[1] Tan Z., Luo X., Xiao L., Tang M., Bode A.M., Dong Z., Cao Y. (2016). The Role of PGC1α in Cancer Metabolism and its Therapeutic Implications. Mol. Cancer Ther..

[2] Villena J.A. (2015). New insights into PGC-1 coactivators: Redefining their role in the regulation of mitochondrial function and beyond. FEBS J..

[3] Lin J., Puigserver P., Donovan J., Tarr P., Spiegelman B.M. (2002). Peroxisome proliferator-activated receptor gamma coactivator 1beta (PGC-1beta), a novel PGC-1-related transcription coactivator associated with host cell factor. J. Biol. Chem..

[4] Jeremic N., Chaturvedi P., Tyagi S.C. (2017). Browning of White Fat: Novel Insight Into Factors, Mechanisms, and Therapeutics. J. Cell. Physiol..

[5] Puigserver P., Spiegelman B.M. (2003). Peroxisome proliferator-activated receptor-gamma coactivator 1 alpha (PGC-1 alpha): Transcriptional coactivator and metabolic regulator. Endocr. Rev..

[6] Chabi B., Adhihetty P.J., Ljubicic V., Hood D.A. (2005). How is mitochondrial biogenesis affected in mitochondrial disease?. Med. Sci. Sports Exerc..

[7] Puigserver P., Wu Z., Park C.W., Graves R., Wright M., Spiegelman B.M. (1998). A Cold-Inducible Coactivator of Nuclear Receptors Linked to Adaptive Thermogenesis. Cell.

[8] Finck B.N., Kelly D.P. (2006). PGC-1 coactivators: Inducible regulators of energy metabolism in health and disease. J. Clin. Investig..

[9] Lin J., Handschin C., Spiegelman B.M. (2005). Metabolic control through the PGC-1 family of transcription coactivators. Cell Metab..

[10] Radak Z., Zhao Z., Koltai E., Ohno H., Atalay M. (2013). Oxygen consumption and usage during physical exercise: The balance between oxidative stress and ROS-dependent adaptive signaling. Antioxid. Redox Signal..

[11] Bost F., Kaminski L. (2019). The metabolic modulator PGC-1α in cancer. Am. J. Cancer Res..

[12] Andersson U., Scarpulla R.C. (2001). Pgc-1-related coactivator, a novel, serum-inducible coactivator of nuclear respiratory factor 1-dependent transcription in mammalian cells. Mol. Cell. Biol..

[13] Jumper J., Evans R., Pritzel A., Green T., Figurnov M., Ronneberger O., Tunyasuvunakool K., Bates R., Žídek A., Potapenko A. (2021). Highly accurate protein structure prediction with AlphaFold. Nature.

[14] Mootha V.K., Bunkenborg J., Olsen J.V., Hjerrild M., Wisniewski J.R., Stahl E., Bolouri M.S., Ray H.N., Sihag S., Kamal M. (2003). Integrated analysis of protein composition, tissue diversity, and gene regulation in mouse mitochondria. Cell.

[15] Kong S., Cai B., Nie Q. (2022). PGC-1α affects skeletal muscle and adipose tissue development by regulating mitochondrial biogenesis. Mol. Genet. Genom..

[16] Wu Z., Puigserver P., Andersson U., Zhang C., Adelmant G., Mootha V., Troy A., Cinti S., Lowell B., Scarpulla R.C. (1999). Mechanisms Controlling Mitochondrial Biogenesis and Respiration through the Thermogenic Coactivator PGC-1. Cell.

[17] Chambers J.M., Wingert R.A. (2020). PGC-1α in Disease: Recent Renal Insights into a Versatile Metabolic Regulator. Cells.

[18] Mouler Rechtman M., Burdelova E.O., Bar-Yishay I., Ben-Yehoyada M., Fishman S., Halpern Z., Shlomai A. (2013). The metabolic regulator PGC-1α links anti-cancer cytotoxic chemotherapy to reactivation of hepatitis B virus. J. Viral Hepat..

[19] Monsalve M., Wu Z., Adelmant G., Puigserver P., Fan M., Spiegelman B.M. (2000). Direct Coupling of Transcription and mRNA Processing through the Thermogenic Coactivator PGC-1. Mol. Cell.

[20] Martínez-Redondo V., Pettersson A.T., Ruas J.L. (2015). The hitchhiker’s guide to PGC-1α isoform structure and biological functions. Diabetologia.

[21] Wang E.T., Sandberg R., Luo S., Khrebtukova I., Zhang L., Mayr C., Kingsmore S.F., Schroth G.P., Burge C.B. (2008). Alternative isoform regulation in human tissue transcriptomes. Nature.

[22] Chinsomboon J., Ruas J., Gupta R.K., Thom R., Shoag J., Rowe G.C., Sawada N., Raghuram S., Arany Z. (2009). The transcriptional coactivator PGC-1alpha mediates exercise-induced angiogenesis in skeletal muscle. Proc. Natl. Acad. Sci. USA.

[23] Miura S., Kai Y., Kamei Y., Ezaki O. (2008). Isoform-specific increases in murine skeletal muscle peroxisome proliferator-activated receptor-gamma coactivator-1alpha (PGC-1alpha) mRNA in response to beta2-adrenergic receptor activation and exercise. Endocrinology.

[24] Yoshioka T., Inagaki K., Noguchi T., Sakai M., Ogawa W., Hosooka T., Iguchi H., Watanabe E., Matsuki Y., Hiramatsu R. (2009). Identification and characterization of an alternative promoter of the human PGC-1alpha gene. Biochem. Biophys. Res. Commun..

[25] Norrbom J., Sällstedt E.K., Fischer H., Sundberg C.J., Rundqvist H., Gustafsson T. (2011). Alternative splice variant PGC-1α-b is strongly induced by exercise in human skeletal muscle. Am. J. Physiol. Endocrinol. Metab..

[26] Ruas J.L., White J.P., Rao R.R., Kleiner S., Brannan K.T., Harrison B.C., Greene N.P., Wu J., Estall J.L., Irving B.A. (2012). A PGC-1α isoform induced by resistance training regulates skeletal muscle hypertrophy. Cell.

[27] Nader G.A., von Walden F., Liu C., Lindvall J., Gutmann L., Pistilli E.E., Gordon P.M. (2014). Resistance exercise training modulates acute gene expression during human skeletal muscle hypertrophy. J. Appl. Physiol..

[28] Tadaishi M., Miura S., Kai Y., Kano Y., Oishi Y., Ezaki O. (2011). Skeletal muscle-specific expression of PGC-1α-b, an exercise-responsive isoform, increases exercise capacity and peak oxygen uptake. PLoS ONE.

[29] Felder T.K., Soyal S.M., Oberkofler H., Hahne P., Auer S., Weiss R., Gadermaier G., Miller K., Krempler F., Esterbauer H. (2011). Characterization of novel peroxisome proliferator-activated receptor γ coactivator-1α (PGC-1α) isoform in human liver. J. Biol. Chem..

[30] Soyal S.M., Felder T.K., Auer S., Hahne P., Oberkofler H., Witting A., Paulmichl M., Landwehrmeyer G.B., Weydt P., Patsch W. (2012). A greatly extended PPARGC1A genomic locus encodes several new brain-specific isoforms and influences Huntington disease age of onset. Hum. Mol. Genet..

[31] Zhang Y., Huypens P., Adamson A.W., Chang J.S., Henagan T.M., Boudreau A., Lenard N.R., Burk D., Klein J., Perwitz N. (2009). Alternative mRNA Splicing Produces a Novel Biologically Active Short Isoform of PGC-1α*. J. Biol. Chem..

[32] Fernandez-Marcos P.J., Auwerx J. (2011). Regulation of PGC-1α, a nodal regulator of mitochondrial biogenesis. Am. J. Clin. Nutr..

[33] Di W., Lv J., Jiang S., Lu C., Yang Z., Ma Z., Hu W., Yang Y., Xu B. (2018). PGC-1: The Energetic Regulator in Cardiac Metabolism. Curr. Issues Mol. Biol..

[34] Suntar I., Sureda A., Belwal T., Sanches Silva A., Vacca R.A., Tewari D., Sobarzo-Sánchez E., Nabavi S.F., Shirooie S., Dehpour A.R. (2020). Natural products, PGC-1α, and Duchenne muscular dystrophy. Acta Pharm. Sin. B.

[35] Cherry A.D., Piantadosi C.A. (2015). Regulation of Mitochondrial Biogenesis and Its Intersection with Inflammatory Responses. Antioxid. Redox Signal..

[36] Hyttinen J., Blasiak J., Tavi P., Kaarniranta K. (2021). Therapeutic potential of PGC-1α in age-related macular degeneration (AMD)—The involvement of mitochondrial quality control, autophagy, and antioxidant response. Expert Opin. Ther. Targets.

[37] Booth F.W., Ruegsegger G.N., Toedebusch R.G., Yan Z. (2015). Endurance Exercise and the Regulation of Skeletal Muscle Metabolism. Prog. Mol. Biol. Transl. Sci..

[38] Marcelo K.L., Means A.R., York B. (2016). The Ca^2+^/Calmodulin/CaMKK2 Axis: Nature’s Metabolic CaMshaft. Trends Endocrinol. Metab..

[39] Potthoff M.J., Wu H., Arnold M.A., Shelton J.M., Backs J., McAnally J., Richardson J.A., Bassel-Duby R., Olson E.N. (2007). Histone deacetylase degradation and MEF2 activation promote the formation of slow-twitch myofibers. J. Clin. Investig..

[40] Sano M., Schneider M.D. (2004). Cyclin-dependent kinase-9: An RNAPII kinase at the nexus of cardiac growth and death cascades. Circ. Res..

[41] Salma N., Song J.S., Arany Z., Fisher D.E. (2015). Transcription Factor Tfe3 Directly Regulates Pgc-1alpha in Muscle. J. Cell. Physiol..

[42] Rius-Pérez S., Torres-Cuevas I., Millán I., Ortega Á.L., Pérez S. (2020). PGC-1α, Inflammation, and Oxidative Stress: An Integrative View in Metabolism. Oxid. Med. Cell. Longev..

[43] Barrès R., Osler M.E., Yan J., Rune A., Fritz T., Caidahl K., Krook A., Zierath J.R. (2009). Non-CpG methylation of the PGC-1alpha promoter through DNMT3B controls mitochondrial density. Cell Metab..

[44] Blasiak J., Pawlowska E., Sobczuk A., Szczepanska J., Kaarniranta K. (2020). The Aging Stress Response and Its Implication for AMD Pathogenesis. Int. J. Mol. Sci..

[45] Chambers K.T., Leone T.C., Sambandam N., Kovacs A., Wagg C.S., Lopaschuk G.D., Finck B.N., Kelly D.P. (2011). Chronic Inhibition of Pyruvate Dehydrogenase in Heart Triggers an Adaptive Metabolic Response*. J. Biol. Chem..

[46] Hardie D.G. (2007). AMP-activated/SNF1 protein kinases: Conserved guardians of cellular energy. Nat. Rev. Mol. Cell Biol..

[47] Parsamanesh N., Asghari A., Sardari S., Tasbandi A., Jamialahmadi T., Xu S., Sahebkar A. (2021). Resveratrol and endothelial function: A literature review. Pharmacol. Res..

[48] Li X., Monks B., Ge Q., Birnbaum M.J. (2007). Akt/PKB regulates hepatic metabolism by directly inhibiting PGC-1alpha transcription coactivator. Nature.

[49] Lustig Y., Ruas J.L., Estall J.L., Lo J.C., Devarakonda S., Laznik D., Choi J.H., Ono H., Olsen J.V., Spiegelman B.M. (2011). Separation of the gluconeogenic and mitochondrial functions of PGC-1{alpha} through S6 kinase. Genes Dev..

[50] Heras-Sandoval D., Pérez-Rojas J.M., Hernández-Damián J., Pedraza-Chaverri J. (2014). The role of PI3K/AKT/mTOR pathway in the modulation of autophagy and the clearance of protein aggregates in neurodegeneration. Cell. Signal..

[51] Whittington H.J., Harding I., Stephenson C.I.M., Bell R., Hausenloy D.J., Mocanu M.M., Yellon D.M. (2013). Cardioprotection in the aging, diabetic heart: The loss of protective Akt signalling. Cardiovasc. Res..

[52] Rodgers J.T., Haas W., Gygi S.P., Puigserver P. (2010). Cdc2-like kinase 2 is an insulin-regulated suppressor of hepatic gluconeogenesis. Cell Metab..

[53] Puigserver P., Rhee J., Donovan J., Walkey C.J., Yoon J.C., Oriente F., Kitamura Y., Altomonte J., Dong H., Accili D. (2003). Insulin-regulated hepatic gluconeogenesis through FOXO1-PGC-1alpha interaction. Nature.

[54] Anderson R.M., Barger J.L., Edwards M.G., Braun K.H., O’Connor C.E., Prolla T.A., Weindruch R. (2008). Dynamic regulation of PGC-1α localization and turnover implicates mitochondrial adaptation in calorie restriction and the stress response. Aging Cell.

[55] Cantó C., Auwerx J. (2009). PGC-1alpha, SIRT1 and AMPK, an energy sensing network that controls energy expenditure. Curr. Opin. Lipidol..

[56] Buck S.W., Gallo C.M., Smith J.S. (2004). Diversity in the Sir2 family of protein deacetylases. J. Leukoc. Biol..

[57] Rodgers J.T., Lerin C., Haas W., Gygi S.P., Spiegelman B.M., Puigserver P. (2005). Nutrient control of glucose homeostasis through a complex of PGC-1alpha and SIRT1. Nature.

[58] Aquilano K., Vigilanza P., Baldelli S., Pagliei B., Rotilio G., Ciriolo M.R. (2010). Peroxisome proliferator-activated receptor gamma co-activator 1alpha (PGC-1alpha) and sirtuin 1 (SIRT1) reside in mitochondria: Possible direct function in mitochondrial biogenesis. J. Biol. Chem..

[59] Aquilano K., Baldelli S., Pagliei B., Ciriolo M.R. (2013). Extranuclear localization of SIRT1 and PGC-1α: An insight into possible roles in diseases associated with mitochondrial dysfunction. Curr. Mol. Med..

[60] Michishita E., Park J.Y., Burneskis J.M., Barrett J.C., Horikawa I. (2005). Evolutionarily conserved and nonconserved cellular localizations and functions of human SIRT proteins. Mol. Biol. Cell.

[61] Cantó C., Jiang L.Q., Deshmukh A.S., Mataki C., Coste A., Lagouge M., Zierath J.R., Auwerx J. (2010). Interdependence of AMPK and SIRT1 for metabolic adaptation to fasting and exercise in skeletal muscle. Cell Metab..

[62] Waldman M., Cohen K., Yadin D., Nudelman V., Gorfil D., Laniado-Schwartzman M., Kornwoski R., Aravot D., Abraham N.G., Arad M. (2018). Regulation of diabetic cardiomyopathy by caloric restriction is mediated by intracellular signaling pathways involving ‘SIRT1 and PGC-1α’. Cardiovasc. Diabetol..

[63] Gerhart-Hines Z., Rodgers J.T., Bare O., Lerin C., Kim S.-H., Mostoslavsky R., Alt F.W., Wu Z., Puigserver P. (2007). Metabolic control of muscle mitochondrial function and fatty acid oxidation through SIRT1/PGC-1α. EMBO J..

[64] Trausch-Azar J., Leone T.C., Kelly D.P., Schwartz A.L. (2010). Ubiquitin Proteasome-dependent Degradation of the Transcriptional Coactivator PGC-1α via the N-terminal Pathway*. J. Biol. Chem..

[65] Olson B.L., Hock M.B., Ekholm-Reed S., Wohlschlegel J.A., Dev K.K., Kralli A., Reed S.I. (2008). SCFCdc4 acts antagonistically to the PGC-1alpha transcriptional coactivator by targeting it for ubiquitin-mediated proteolysis. Genes Dev..

[66] Rytinki M.M., Palvimo J.J. (2009). SUMOylation Attenuates the Function of PGC-1α*. J. Biol. Chem..

[67] Cai R., Yu T., Huang C., Xia X., Liu X., Gu J., Xue S., Yeh E.T., Cheng J. (2012). SUMO-specific Protease 1 Regulates Mitochondrial Biogenesis through PGC-1α*. J. Biol. Chem..

[68] Teyssier C., Ma H., Emter R., Kralli A., Stallcup M.R. (2005). Activation of nuclear receptor coactivator PGC-1alpha by arginine methylation. Genes Dev..

[69] Ruan H.-B., Han X., Li M.-D., Singh J.P., Qian K., Azarhoush S., Zhao L., Bennett A.M., Samuel V.T., Wu J. (2012). O-GlcNAc transferase/host cell factor C1 complex regulates gluconeogenesis by modulating PGC-1α stability. Cell Metab..

[70] Housley M.P., Udeshi N.D., Rodgers J.T., Shabanowitz J., Puigserver P., Hunt D.F., Hart G.W. (2009). A PGC-1alpha-O-GlcNAc transferase complex regulates FoxO transcription factor activity in response to glucose. J. Biol. Chem..

[71] Luo X., Liao C., Quan J., Cheng C., Zhao X., Bode A.M., Cao Y. (2019). Posttranslational regulation of PGC-1α and its implication in cancer metabolism. Int. J. Cancer.

[72] Halling J.F., Pilegaard H. (2020). PGC-1α-mediated regulation of mitochondrial function and physiological implications. Appl. Physiol. Nutr. Metab..

[73] Fontecha-Barriuso M., Martin-Sanchez D., Martinez-Moreno J.M., Monsalve M., Ramos A.M., Sanchez-Niño M.D., Ruiz-Ortega M., Ortiz A., Sanz A.B. (2020). The Role of PGC-1α and Mitochondrial Biogenesis in Kidney Diseases. Biomolecules.

[74] Diaz-Gerevini G.T., Repossi G., Dain A., Tarres M.C., Das U.N., Eynard A.R. (2016). Beneficial action of resveratrol: How and why?. Nutrition.

[75] Ray Hamidie R.D., Yamada T., Ishizawa R., Saito Y., Masuda K. (2015). Curcumin treatment enhances the effect of exercise on mitochondrial biogenesis in skeletal muscle by increasing cAMP levels. Metabolism.

[76] Wang Y., Zhao X., Lotz M., Terkeltaub R., Liu-Bryan R. (2015). Mitochondrial biogenesis is impaired in osteoarthritis chondrocytes but reversible via peroxisome proliferator-activated receptor γ coactivator 1α. Arthritis Rheumatol..

[77] Vásquez-Reyes S., Velázquez-Villegas L.A., Vargas-Castillo A., Noriega L.G., Torres N., Tovar A.R. (2021). Dietary bioactive compounds as modulators of mitochondrial function. J. Nutr. Biochem..

[78] Maissan P., Mooij E.J., Barberis M. (2021). Sirtuins-Mediated System-Level Regulation of Mammalian Tissues at the Interface between Metabolism and Cell Cycle: A Systematic Review. Biology.

[79] Ventura-Clapier R., Moulin M., Piquereau J., Lemaire C., Mericskay M., Veksler V., Garnier A. (2017). Mitochondria: A central target for sex differences in pathologies. Clin. Sci..

[80] Scarpulla R.C. (2008). Transcriptional paradigms in mammalian mitochondrial biogenesis and function. Physiol. Rev..

[81] Irrcher I., Ljubicic V., Kirwan A.F., Hood D.A. (2008). AMP-activated protein kinase-regulated activation of the PGC-1alpha promoter in skeletal muscle cells. PLoS ONE.

[82] Olesen J., Kiilerich K., Pilegaard H. (2010). PGC-1alpha-mediated adaptations in skeletal muscle. Pflugers Arch..

[83] Blesa J.R., Hernández-Yago J. (2006). Distinct functional contributions of 2 GABP-NRF-2 recognition sites within the context of the human TOMM70 promoter. Biochem. Cell Biol..

[84] Onyango I.G., Bennett J.P., Stokin G.B. (2021). Regulation of neuronal bioenergetics as a therapeutic strategy in neurodegenerative diseases. Neural Regen. Res..

[85] Ross J.A., Pearson A., Levy Y., Cardel B., Handschin C., Ochala J. (2017). Exploring the Role of PGC-1α in Defining Nuclear Organisation in Skeletal Muscle Fibres. J. Cell. Physiol..

[86] Brüser C., Keller-Findeisen J., Jakobs S. (2021). The TFAM-to-mtDNA ratio defines inner-cellular nucleoid populations with distinct activity levels. Cell Rep..

[87] Taherzadeh-Fard E., Saft C., Akkad D.A., Wieczorek S., Haghikia A., Chan A., Epplen J.T., Arning L. (2011). PGC-1alpha downstream transcription factors NRF-1 and TFAM are genetic modifiers of Huntington disease. Mol. Neurodegener..

[88] Tatsuta T., Langer T. (2008). Quality control of mitochondria: Protection against neurodegeneration and ageing. EMBO J..

[89] Kim Y., Triolo M., Hood D.A. (2017). Impact of Aging and Exercise on Mitochondrial Quality Control in Skeletal Muscle. Oxid. Med. Cell. Longev..

[90] Zhang Q., Lei Y.-H., Zhou J.-P., Hou Y.-Y., Wan Z., Wang H.-L., Meng H. (2019). Role of PGC-1α in Mitochondrial Quality Control in Neurodegenerative Diseases. Neurochem. Res..

[91] Chen H., Vermulst M., Wang Y.E., Chomyn A., Prolla T.A., McCaffery J.M., Chan D.C. (2010). Mitochondrial fusion is required for mtDNA stability in skeletal muscle and tolerance of mtDNA mutations. Cell.

[92] Cipolat S., Martins de Brito O., Dal Zilio B., Scorrano L. (2004). OPA1 requires mitofusin 1 to promote mitochondrial fusion. Proc. Natl. Acad. Sci. USA.

[93] Losón O.C., Song Z., Chen H., Chan D.C. (2013). Fis1, Mff, MiD49, and MiD51 mediate Drp1 recruitment in mitochondrial fission. Mol. Biol. Cell.

[94] Mai S., Klinkenberg M., Auburger G., Bereiter-Hahn J., Jendrach M. (2010). Decreased expression of Drp1 and Fis1 mediates mitochondrial elongation in senescent cells and enhances resistance to oxidative stress through PINK1. J. Cell Sci..

[95] Peng K., Yang L., Wang J., Ye F., Dan G., Zhao Y., Cai Y., Cui Z., Ao L., Liu J. (2017). The Interaction of Mitochondrial Biogenesis and Fission/Fusion Mediated by PGC-1α Regulates Rotenone-Induced Dopaminergic Neurotoxicity. Mol. Neurobiol..

[96] Dabrowska A., Venero J.L., Iwasawa R., Hankir M., Rahman S., Boobis A., Hajji N. (2015). PGC-1α controls mitochondrial biogenesis and dynamics in lead-induced neurotoxicity. Aging.

[97] Chuang Y.-C., Lin T.-K., Yang D.-I., Yang J.-L., Liou C.-W., Chen S.-D. (2016). Peroxisome proliferator-activated receptor-gamma dependent pathway reduces the phosphorylation of dynamin-related protein 1 and ameliorates hippocampal injury induced by global ischemia in rats. J. Biomed. Sci..

[98] Zhang Z., Zhang X., Meng L., Gong M., Li J., Shi W., Qiu J., Yang Y., Zhao J., Suo Y. (2021). Pioglitazone Inhibits Diabetes-Induced Atrial Mitochondrial Oxidative Stress and Improves Mitochondrial Biogenesis, Dynamics, and Function Through the PPAR-γ/PGC-1α Signaling Pathway. Front. Pharmacol..

[99] Martin O.J., Lai L., Soundarapandian M.M., Leone T.C., Zorzano A., Keller M.P., Attie A.D., Muoio D.M., Kelly D.P. (2014). A role for peroxisome proliferator-activated receptor γ coactivator-1 in the control of mitochondrial dynamics during postnatal cardiac growth. Circ. Res..

[100] Zhu J., Wang K.Z.Q., Chu C.T. (2013). After the banquet: Mitochondrial biogenesis, mitophagy, and cell survival. Autophagy.

[101] Mizushima N., Komatsu M. (2011). Autophagy: Renovation of cells and tissues. Cell.

[102] Zorova L.D., Popkov V.A., Plotnikov E.Y., Silachev D.N., Pevzner I.B., Jankauskas S.S., Babenko V.A., Zorov S.D., Balakireva A.V., Juhaszova M. (2018). Mitochondrial membrane potential. Anal. Biochem..

[103] Palikaras K., Tavernarakis N. (2014). Mitochondrial homeostasis: The interplay between mitophagy and mitochondrial biogenesis. Exp. Gerontol..

[104] Valle I., Alvarez-Barrientos A., Arza E., Lamas S., Monsalve M. (2005). PGC-1alpha regulates the mitochondrial antioxidant defense system in vascular endothelial cells. Cardiovasc. Res..

[105] Zhou L., Pinho R., Gu Y., Radak Z. (2022). The Role of SIRT3 in Exercise and Aging. Cells.

[106] Xu L., Li Y., Zhou L., Dorfman R.G., Liu L., Cai R., Jiang C., Tang D., Wang Y., Zou X. (2019). SIRT3 elicited an anti-Warburg effect through HIF1α/PDK1/PDHA1 to inhibit cholangiocarcinoma tumorigenesis. Cancer Med..

[107] O’Flaherty C. (2019). Orchestrating the antioxidant defenses in the epididymis. Andrology.

[108] Cadenas S. (2018). Mitochondrial uncoupling, ROS generation and cardioprotection. Biochim. Biophys. Acta (BBA)-Bioenerg..

[109] Toda C., Diano S. (2014). Mitochondrial UCP2 in the central regulation of metabolism. Best Pract. Res. Clin. Endocrinol. Metab..

[110] Pérez S., Rius-Pérez S., Finamor I., Martí-Andrés P., Prieto I., García R., Monsalve M., Sastre J. (2019). Obesity causes PGC-1α deficiency in the pancreas leading to marked IL-6 upregulation via NF-κB in acute pancreatitis. J. Pathol..

[111] Qi Y., Yin X., Wang S., Jiang H., Wang X., Ren M., Su X.-P., Lei S., Feng H. (2015). PGC-1α Silencing Compounds the Perturbation of Mitochondrial Function Caused by Mutant SOD1 in Skeletal Muscle of ALS Mouse Model. Front. Aging Neurosci..

[112] Alvarez-Guardia D., Palomer X., Coll T., Davidson M.M., Chan T.O., Feldman A.M., Laguna J.C., Vázquez-Carrera M. (2010). The p65 subunit of NF-kappaB binds to PGC-1alpha, linking inflammation and metabolic disturbances in cardiac cells. Cardiovasc. Res..

[113] Chen S.-D., Yang D.-I., Lin T.-K., Shaw F.-Z., Liou C.-W., Chuang Y.-C. (2011). Roles of oxidative stress, apoptosis, PGC-1α and mitochondrial biogenesis in cerebral ischemia. Int. J. Mol. Sci..

[114] Kadlec A.O., Chabowski D.S., Ait-Aissa K., Gutterman D.D. (2016). Role of PGC-1α in Vascular Regulation: Implications for Atherosclerosis. Arterioscler. Thromb. Vasc. Biol..

[115] Eisele P.S., Handschin C. (2014). Functional crosstalk of PGC-1 coactivators and inflammation in skeletal muscle pathophysiology. Semin. Immunopathol..

[116] Eleutherio E.C.A., Silva Magalhães R.S., de Araújo Brasil A., Monteiro Neto J.R., de Holanda Paranhos L. (2021). SOD1, more than just an antioxidant. Arch. Biochem. Biophys..

[117] Chen Y.-I., Wei P.-C., Hsu J.-L., Su F.-Y., Lee W.-H. (2016). NPGPx (GPx7): A novel oxidative stress sensor/transmitter with multiple roles in redox homeostasis. Am. J. Transl. Res..

[118] Remels A.H.V., Gosker H.R., Bakker J., Guttridge D.C., Schols A.M.W.J., Langen R.C.J. (2013). Regulation of skeletal muscle oxidative phenotype by classical NF-κB signalling. Biochim. Biophys. Acta.

[119] Tran M., Tam D., Bardia A., Bhasin M., Rowe G.C., Kher A., Zsengeller Z.K., Akhavan-Sharif M.R., Khankin E.V., Saintgeniez M. (2011). PGC-1α promotes recovery after acute kidney injury during systemic inflammation in mice. J. Clin. Investig..

[120] Kim M.S., Shigenaga J.K., Moser A.H., Feingold K.R., Grunfeld C. (2005). Suppression of estrogen-related receptor alpha and medium-chain acyl-coenzyme A dehydrogenase in the acute-phase response. J. Lipid Res..

[121] Yu X.X., Barger J.L., Boyer B.B., Brand M.D., Pan G., Adams S.H. (2000). Impact of endotoxin on UCP homolog mRNA abundance, thermoregulation, and mitochondrial proton leak kinetics. Am. J. Physiol. Endocrinol. Metab..

[122] Wu Z., Berlemann L.A., Bader V., Sehr D.A., Dawin E., Covallero A., Meschede J., Angersbach L., Showkat C., Michaelis J.B. (2022). LUBAC assembles a ubiquitin signaling platform at mitochondria for signal amplification and transport of NF-κB to the nucleus. EMBO J..

[123] Kang C., Ji L.L. (2012). Role of PGC-1α signaling in skeletal muscle health and disease. Ann. N. Y. Acad. Sci..

[124] Yuan S., Liu X., Zhu X., Qu Z., Gong Z., Li J., Xiao L., Yang Y., Liu H., Sun L. (2018). The Role of TLR4 on PGC-1α-Mediated Oxidative Stress in Tubular Cell in Diabetic Kidney Disease. Oxid. Med. Cell. Longev..

[125] Besse-Patin A., Léveillé M., Oropeza D., Nguyen B.N., Prat A., Estall J.L. (2017). Estrogen Signals Through Peroxisome Proliferator-Activated Receptor-γ Coactivator 1α to Reduce Oxidative Damage Associated With Diet-Induced Fatty Liver Disease. Gastroenterology.

[126] Baldelli S., Aquilano K., Ciriolo M.R. (2014). PGC-1α buffers ROS-mediated removal of mitochondria during myogenesis. Cell Death Dis..

[127] Eisele P.S., Furrer R., Beer M., Handschin C. (2015). The PGC-1 coactivators promote an anti-inflammatory environment in skeletal muscle in vivo. Biochem. Biophys. Res. Commun..

[128] Fontecha-Barriuso M., Martín-Sánchez D., Martinez-Moreno J.M., Carrasco S., Ruiz-Andrés O., Monsalve M., Sanchez-Ramos C., Gómez M.J., Ruiz-Ortega M., Sánchez-Niño M.D. (2019). PGC-1α deficiency causes spontaneous kidney inflammation and increases the severity of nephrotoxic AKI. J. Pathol..

[129] Quirós P.M., Mottis A., Auwerx J. (2016). Mitonuclear communication in homeostasis and stress. Nat. Rev. Mol. Cell Biol..

[130] St-Pierre J., Drori S., Uldry M., Silvaggi J.M., Rhee J., Jäger S., Handschin C., Zheng K., Lin J., Yang W. (2006). Suppression of reactive oxygen species and neurodegeneration by the PGC-1 transcriptional coactivators. Cell.

[131] Keller J.N., Kindy M.S., Holtsberg F.W., St Clair D.K., Yen H.C., Germeyer A., Steiner S.M., Bruce-Keller A.J., Hutchins J.B., Mattson M.P. (1998). Mitochondrial manganese superoxide dismutase prevents neural apoptosis and reduces ischemic brain injury: Suppression of peroxynitrite production, lipid peroxidation, and mitochondrial dysfunction. J. Neurosci..

[132] Noshita N., Sugawara T., Fujimura M., Morita-Fujimura Y., Chan P.H. (2001). Manganese Superoxide Dismutase Affects Cytochrome c Release and Caspase-9 Activation After Transient Focal Cerebral Ischemia in Mice. J. Cereb. Blood Flow Metab..

[133] Dasgupta D., Mahadev Bhat S., Price A.L., Delmotte P., Sieck G.C. (2023). Molecular Mechanisms Underlying TNFα-Induced Mitochondrial Biogenesis in Human Airway Smooth Muscle. Int. J. Mol. Sci..

[134] Evans M.J., Scarpulla R.C. (1990). NRF-1: A trans-activator of nuclear-encoded respiratory genes in animal cells. Genes Dev..

[135] Guarás A., Perales-Clemente E., Calvo E., Acín-Pérez R., Loureiro-Lopez M., Pujol C., Martínez-Carrascoso I., Nuñez E., García-Marqués F., Rodríguez-Hernández M.A. (2016). The CoQH2/CoQ Ratio Serves as a Sensor of Respiratory Chain Efficiency. Cell Rep..

[136] Hüttemann M., Pecina P., Rainbolt M., Sanderson T.H., Kagan V.E., Samavati L., Doan J.W., Lee I. (2011). The multiple functions of cytochrome c and their regulation in life and death decisions of the mammalian cell: From respiration to apoptosis. Mitochondrion.

[137] Murphy M.P. (2009). How mitochondria produce reactive oxygen species. Biochem. J..

[138] Hirst J., King M.S., Pryde K.R. (2008). The production of reactive oxygen species by complex I. Biochem. Soc. Trans..

[139] Virbasius J.V., Scarpulla R.C. (1994). Activation of the human mitochondrial transcription factor A gene by nuclear respiratory factors: A potential regulatory link between nuclear and mitochondrial gene expression in organelle biogenesis. Proc. Natl. Acad. Sci. USA.

[140] Gleyzer N., Vercauteren K., Scarpulla R.C. (2005). Control of mitochondrial transcription specificity factors (TFB1M and TFB2M) by nuclear respiratory factors (NRF-1 and NRF-2) and PGC-1 family coactivators. Mol. Cell. Biol..

[141] Kukat C., Wurm C.A., Spåhr H., Falkenberg M., Larsson N.-G., Jakobs S. (2011). Super-resolution microscopy reveals that mammalian mitochondrial nucleoids have a uniform size and frequently contain a single copy of mtDNA. Proc. Natl. Acad. Sci. USA.

[142] Ngo H.B., Lovely G.A., Phillips R., Chan D.C. (2014). Distinct structural features of TFAM drive mitochondrial DNA packaging versus transcriptional activation. Nat. Commun..

[143] Matsushima Y., Matsumura K., Ishii S., Inagaki H., Suzuki T., Matsuda Y., Beck K., Kitagawa Y. (2003). Functional domains of chicken mitochondrial transcription factor A for the maintenance of mitochondrial DNA copy number in lymphoma cell line DT40. J. Biol. Chem..

[144] Kanki T., Ohgaki K., Gaspari M., Gustafsson C.M., Fukuoh A., Sasaki N., Hamasaki N., Kang D. (2004). Architectural role of mitochondrial transcription factor A in maintenance of human mitochondrial DNA. Mol. Cell. Biol..

[145] Scheffler I.E. (2008). Mitochondria.

[146] Berger J., Moller D.E. (2002). The mechanisms of action of PPARs. Annu. Rev. Med..

[147] Tanaka T., Yamamoto J., Iwasaki S., Asaba H., Hamura H., Ikeda Y., Watanabe M., Magoori K., Ioka R.X., Tachibana K. (2003). Activation of peroxisome proliferator-activated receptor delta induces fatty acid beta-oxidation in skeletal muscle and attenuates metabolic syndrome. Proc. Natl. Acad. Sci. USA.

[148] Fan W., Evans R. (2015). PPARs and ERRs: Molecular mediators of mitochondrial metabolism. Curr. Opin. Cell Biol..

[149] Hüttemann M., Lee I., Samavati L., Yu H., Doan J.W. (2007). Regulation of mitochondrial oxidative phosphorylation through cell signaling. Biochim. Biophys. Acta.

[150] Eckel R.H., Grundy S.M., Zimmet P.Z. (2005). The metabolic syndrome. Lancet.

[151] Vandenbeek R., Khan N.P., Estall J.L. (2018). Linking Metabolic Disease With the PGC-1α Gly482Ser Polymorphism. Endocrinology.

[152] Lee G., Uddin M.J., Kim Y., Ko M., Yu I., Ha H. (2019). PGC-1α, a potential therapeutic target against kidney aging. Aging Cell.

[153] Sahin E., Colla S., Liesa M., Moslehi J., Müller F.L., Guo M., Cooper M., Kotton D., Fabian A.J., Walkey C. (2011). Telomere dysfunction induces metabolic and mitochondrial compromise. Nature.

[154] Kang Y., Zhang H., Zhao Y., Wang Y., Wang W., He Y., Zhang W., Zhang W., Zhu X., Zhou Y. (2018). Telomere Dysfunction Disturbs Macrophage Mitochondrial Metabolism and the NLRP3 Inflammasome through the PGC-1α/TNFAIP3 Axis. Cell Rep..

[155] Xiong S., Patrushev N., Forouzandeh F., Hilenski L., Alexander R.W. (2015). PGC-1α Modulates Telomere Function and DNA Damage in Protecting against Aging-Related Chronic Diseases. Cell Rep..

[156] Sczelecki S., Besse-Patin A., Abboud A., Kleiner S., Laznik-Bogoslavski D., Wrann C.D., Ruas J.L., Haibe-Kains B., Estall J.L. (2014). Loss of Pgc-1α expression in aging mouse muscle potentiates glucose intolerance and systemic inflammation. Am. J. Physiol. Endocrinol. Metab..

[157] Choi C.S., Befroy D.E., Codella R., Kim S., Reznick R.M., Hwang Y.-J., Liu Z.-X., Lee H.-Y., Distefano A., Samuel V.T. (2008). Paradoxical effects of increased expression of PGC-1alpha on muscle mitochondrial function and insulin-stimulated muscle glucose metabolism. Proc. Natl. Acad. Sci. USA.

[158] Conklin K.A. (2004). Chemotherapy-associated oxidative stress: Impact on chemotherapeutic effectiveness. Integr. Cancer Ther..

[159] Leach J.K., van Tuyle G., Lin P.S., Schmidt-Ullrich R., Mikkelsen R.B. (2001). Ionizing radiation-induced, mitochondria-dependent generation of reactive oxygen/nitrogen. Cancer Res..

[160] Liou G.-Y., Storz P. (2010). Reactive oxygen species in cancer. Free Radic. Res..

[161] Dirat B., Ader I., Golzio M., Massa F., Mettouchi A., Laurent K., Larbret F., Malavaud B., Cormont M., Lemichez E. (2015). Inhibition of the GTPase Rac1 mediates the antimigratory effects of metformin in prostate cancer cells. Mol. Cancer Ther..

[162] Laurent V., Guérard A., Mazerolles C., Le Gonidec S., Toulet A., Nieto L., Zaidi F., Majed B., Garandeau D., Socrier Y. (2016). Periprostatic adipocytes act as a driving force for prostate cancer progression in obesity. Nat. Commun..

[163] Wang Y.Y., Attané C., Milhas D., Dirat B., Dauvillier S., Guerard A., Gilhodes J., Lazar I., Alet N., Laurent V. (2017). Mammary adipocytes stimulate breast cancer invasion through metabolic remodeling of tumor cells. JCI Insight.

[164] D’Errico I., Salvatore L., Murzilli S., Lo Sasso G., Latorre D., Martelli N., Egorova A.V., Polishuck R., Madeyski-Bengtson K., Lelliott C. (2011). Peroxisome proliferator-activated receptor-gamma coactivator 1-alpha (PGC1alpha) is a metabolic regulator of intestinal epithelial cell fate. Proc. Natl. Acad. Sci. USA.

[165] LaGory E.L., Wu C., Taniguchi C.M., Ding C.-K.C., Chi J.-T., von Eyben R., Scott D.A., Richardson A.D., Giaccia A.J. (2015). Suppression of PGC-1α Is Critical for Reprogramming Oxidative Metabolism in Renal Cell Carcinoma. Cell Rep..

[166] Wang X., Moraes C.T. (2011). Increases in mitochondrial biogenesis impair carcinogenesis at multiple levels. Mol. Oncol..

[167] Burchard J., Zhang C., Liu A.M., Poon R.T.P., Lee N.P.Y., Wong K.-F., Sham P.C., Lam B.Y., Ferguson M.D., Tokiwa G. (2010). microRNA-122 as a regulator of mitochondrial metabolic gene network in hepatocellular carcinoma. Mol. Syst. Biol..

[168] Zhang Y., Ba Y., Liu C., Sun G., Ding L., Gao S., Hao J., Yu Z., Zhang J., Zen K. (2007). PGC-1alpha induces apoptosis in human epithelial ovarian cancer cells through a PPARgamma-dependent pathway. Cell Res..

[169] Petrocelli J.J., Drummond M.J. (2020). PGC-1α-Targeted Therapeutic Approaches to Enhance Muscle Recovery in Aging. Int. J. Environ. Res. Public Health.

[170] Pezzuto J.M. (2019). Resveratrol: Twenty Years of Growth, Development and Controversy. Biomol. Ther..

[171] Pannu N., Bhatnagar A. (2019). Resveratrol: From enhanced biosynthesis and bioavailability to multitargeting chronic diseases. Biomed. Pharmacother..

[172] Niu W., Wang H., Wang B., Mao X., Du M. (2021). Resveratrol improves muscle regeneration in obese mice through enhancing mitochondrial biogenesis. J. Nutr. Biochem..

[173] Kim Y., Park C.W. (2016). Adenosine monophosphate-activated protein kinase in diabetic nephropathy. Kidney Res. Clin. Pract..

[174] Barzilai N., Crandall J.P., Kritchevsky S.B., Espeland M.A. (2016). Metformin as a Tool to Target Aging. Cell Metab..

[175] Owen M.R., Doran E., Halestrap A.P. (2000). Evidence that metformin exerts its anti-diabetic effects through inhibition of complex 1 of the mitochondrial respiratory chain. Biochem. J..

[176] El-Mir M.Y., Nogueira V., Fontaine E., Avéret N., Rigoulet M., Leverve X. (2000). Dimethylbiguanide inhibits cell respiration via an indirect effect targeted on the respiratory chain complex I. J. Biol. Chem..

[177] Cao J., Meng S., Chang E., Beckwith-Fickas K., Xiong L., Cole R.N., Radovick S., Wondisford F.E., He L. (2014). Low concentrations of metformin suppress glucose production in hepatocytes through AMP-activated protein kinase (AMPK). J. Biol. Chem..

[178] LaMoia T.E., Butrico G.M., Kalpage H.A., Goedeke L., Hubbard B.T., Vatner D.F., Gaspar R.C., Zhang X.-M., Cline G.W., Nakahara K. (2022). Metformin, phenformin, and galegine inhibit complex IV activity and reduce glycerol-derived gluconeogenesis. Proc. Natl. Acad. Sci. USA.

[179] Graham G.G., Punt J., Arora M., Day R.O., Doogue M.P., Duong J.K., Furlong T.J., Greenfield J.R., Greenup L.C., Kirkpatrick C.M. (2011). Clinical pharmacokinetics of metformin. Clin. Pharmacokinet..

[180] Timmins P., Donahue S., Meeker J., Marathe P. (2005). Steady-state pharmacokinetics of a novel extended-release metformin formulation. Clin. Pharmacokinet..

[181] LaMoia T.E., Shulman G.I. (2021). Cellular and Molecular Mechanisms of Metformin Action. Endocr. Rev..

[182] Madiraju A.K., Qiu Y., Perry R.J., Rahimi Y., Zhang X.-M., Zhang D., Camporez J.-P.G., Cline G.W., Butrico G.M., Kemp B.E. (2018). Metformin inhibits gluconeogenesis via a redox-dependent mechanism in vivo. Nat. Med..

[183] Madiraju A.K., Erion D.M., Rahimi Y., Zhang X.-M., Braddock D.T., Albright R.A., Prigaro B.J., Wood J.L., Bhanot S., MacDonald M.J. (2014). Metformin suppresses gluconeogenesis by inhibiting mitochondrial glycerophosphate dehydrogenase. Nature.

[184] Anisimov V.N., Berstein L.M., Popovich I.G., Zabezhinski M.A., Egormin P.A., Piskunova T.S., Semenchenko A.V., Tyndyk M.L., Yurova M.N., Kovalenko I.G. (2011). If started early in life, metformin treatment increases life span and postpones tumors in female SHR mice. Aging.

[185] Anisimov V.N., Zabezhinski M.A., Popovich I.G., Piskunova T.S., Semenchenko A.V., Tyndyk M.L., Yurova M.N., Antoch M.P., Blagosklonny M.V. (2010). Rapamycin extends maximal lifespan in cancer-prone mice. Am. J. Pathol..

[186] Fischer K.E., Gelfond J.A.L., Soto V.Y., Han C., Someya S., Richardson A., Austad S.N. (2015). Health Effects of Long-Term Rapamycin Treatment: The Impact on Mouse Health of Enteric Rapamycin Treatment from Four Months of Age throughout Life. PLoS ONE.

[187] Harrison D.E., Strong R., Sharp Z.D., Nelson J.F., Astle C.M., Flurkey K., Nadon N.L., Wilkinson J.E., Frenkel K., Carter C.S. (2009). Rapamycin fed late in life extends lifespan in genetically heterogeneous mice. Nature.

[188] Hurez V., Dao V., Liu A., Pandeswara S., Gelfond J., Sun L., Bergman M., Orihuela C.J., Galvan V., Padrón Á. (2015). Chronic mTOR inhibition in mice with rapamycin alters T, B, myeloid, and innate lymphoid cells and gut flora and prolongs life of immune-deficient mice. Aging Cell.

[189] Miller R.A., Harrison D.E., Astle C.M., Fernandez E., Flurkey K., Han M., Javors M.A., Li X., Nadon N.L., Nelson J.F. (2014). Rapamycin-mediated lifespan increase in mice is dose and sex dependent and metabolically distinct from dietary restriction. Aging Cell.

[190] Ramos F.J., Chen S.C., Garelick M.G., Dai D.-F., Liao C.-Y., Schreiber K.H., MacKay V.L., An E.H., Strong R., Ladiges W.C. (2012). Rapamycin reverses elevated mTORC1 signaling in lamin A/C-deficient mice, rescues cardiac and skeletal muscle function, and extends survival. Sci. Transl. Med..

[191] Wu J.J., Liu J., Chen E.B., Wang J.J., Cao L., Narayan N., Fergusson M.M., Rovira I.I., Allen M., Springer D.A. (2013). Increased mammalian lifespan and a segmental and tissue-specific slowing of aging after genetic reduction of mTOR expression. Cell Rep..

[192] Chen G., Chen H., Wang C., Peng Y., Sun L., Liu H., Liu F. (2012). Rapamycin ameliorates kidney fibrosis by inhibiting the activation of mTOR signaling in interstitial macrophages and myofibroblasts. PLoS ONE.

[193] Cunningham J.T., Rodgers J.T., Arlow D.H., Vazquez F., Mootha V.K., Puigserver P. (2007). mTOR controls mitochondrial oxidative function through a YY1-PGC-1alpha transcriptional complex. Nature.

[194] Wang X., Huang N., Yang M., Wei D., Tai H., Han X., Gong H., Zhou J., Qin J., Wei X. (2017). FTO is required for myogenesis by positively regulating mTOR-PGC-1α pathway-mediated mitochondria biogenesis. Cell Death Dis..

[195] Kim E.N., Lim J.H., Kim M.Y., Kim H.W., Park C.W., Chang Y.S., Choi B.S. (2016). PPARα agonist, fenofibrate, ameliorates age-related renal injury. Exp. Gerontol..

[196] Dillon L.M., Hida A., Garcia S., Prolla T.A., Moraes C.T. (2012). Long-term bezafibrate treatment improves skin and spleen phenotypes of the mtDNA mutator mouse. PLoS ONE.

[197] Da Rosa-Junior N.T., Parmeggiani B., Glänzel N.M., de Moura Alvorcem L., Brondani M., Britto R., Grings M., Ortiz V.D., Turck P., Da Rosa Araujo A.S. (2022). Antioxidant system disturbances and mitochondrial dysfunction induced by 3-methyglutaric acid in rat heart are prevented by bezafibrate. Eur. J. Pharmacol..

[198] Zhang L., Liu J., Zhou F., Wang W., Chen N. (2018). PGC-1α ameliorates kidney fibrosis in mice with diabetic kidney disease through an antioxidative mechanism. Mol. Med. Rep..

[199] Wang H., Yan W.-J., Zhang J.-L., Zhang F.-Y., Gao C., Wang Y.-J., Bond Law W., Tao L. (2017). Adiponectin partially rescues high glucose/high fat-induced impairment of mitochondrial biogenesis and function in a PGC-1α dependent manner. Eur. Rev. Med. Pharmacol. Sci..

[200] Yan W., Zhang H., Liu P., Wang H., Liu J., Gao C., Liu Y., Lian K., Yang L., Sun L. (2013). Impaired mitochondrial biogenesis due to dysfunctional adiponectin-AMPK-PGC-1α signaling contributing to increased vulnerability in diabetic heart. Basic Res. Cardiol..

[201] Popov L.-D. (2020). Mitochondrial biogenesis: An update. J. Cell. Mol. Med..

[202] Jiang S., Teague A.M., Tryggestad J.B., Chernausek S.D. (2017). Role of microRNA-130b in placental PGC-1α/TFAM mitochondrial biogenesis pathway. Biochem. Biophys. Res. Commun..

[203] Gorska-Ponikowska M., Kuban-Jankowska A., Eisler S.A., Perricone U., Lo Bosco G., Barone G., Nussberger S. (2018). 2-Methoxyestradiol Affects Mitochondrial Biogenesis Pathway and Succinate Dehydrogenase Complex Flavoprotein Subunit A in Osteosarcoma Cancer Cells. Cancer Genom. Proteom..

[204] Valerio A., Cardile A., Cozzi V., Bracale R., Tedesco L., Pisconti A., Palomba L., Cantoni O., Clementi E., Moncada S. (2006). TNF-alpha downregulates eNOS expression and mitochondrial biogenesis in fat and muscle of obese rodents. J. Clin. Investig..

[205] Boudina S., Sena S., O’Neill B.T., Tathireddy P., Young M.E., Abel E.D. (2005). Reduced mitochondrial oxidative capacity and increased mitochondrial uncoupling impair myocardial energetics in obesity. Circulation.

[206] Qi R., Wang D., Xing L., Wu Z. (2018). Cyclosporin A inhibits mitochondrial biogenesis in Hep G2 cells. Biochem. Biophys. Res. Commun..

